# Hyaluronic Acid Hydrogel Implants for Sustained Release of Oxaliplatin and Resiquimod to Prevent Hepatocellular Carcinoma Recurrence Post‐Radiofrequency Ablation

**DOI:** 10.1002/advs.202509309

**Published:** 2025-09-30

**Authors:** Yuezhan Shan, Hongyu Chu, Sheyu Ye, Guofeng Ji, Jiayi Zhao, Xinghui Si, Yumin Zhong, Youmao Tao, Jingwei Shi, Xuedong Fang

**Affiliations:** ^1^ Department of Gastrointestinal and Colorectal Surgery China‐Japan Union Hospital of Jilin University Changchun 130033 China; ^2^ Department of Laboratory Medicine Center China‐Japan Union Hospital Jilin University Changchun 130033 China; ^3^ Department of General Surgery Xuanwu Hospital Capital Medical University Beijing 100053 China; ^4^ Key Laboratory of Polymer Ecomaterials Changchun Institute of Applied Chemistry Chinese Academy of Sciences Changchun 130022 China; ^5^ North Sichuan Medical College Sichuan 637199 China

**Keywords:** biopolymer implants, hyaluronic acid, oxaliplatin, radiofrequency ablation, TLR7/8 agonist

## Abstract

Hepatocellular carcinoma (HCC) remains a global challenge due to limited therapies and high recurrence. While radiofrequency ablation (RFA) is commonly used, its efficacy is hindered by incomplete ablation. Local immunotherapy has gained attention as a novel strategy to improve therapeutic efficacy through targeted activation of anti‐tumor immunity. In this work, a biopolymer implant (BI) hydrogel based on hyaluronic acid is developed for the sustained release of oxaliplatin (OXA) and resiquimod (R848), aiming to enhance the immunotherapeutic efficacy of RFA in liver cancer. The injectable BI hydrogel, formed via Schiff base linkage, enables prolonged drug release and exhibits favorable biosafety. In subcutaneous HCC models, RFA + BI(OXA + R848) achieved 97.1% tumor inhibition and 40% complete remission. Additionally, the combination therapy is further validated in bilateral tumor and orthotopic HCC models, demonstrating superior tumor suppression. Furthermore, flow cytometry and RNA sequencing confirmed that RFA combined with BI(OXA + R848) significantly enhanced intratumoral dendritic cell activation, promoted the recruitment of CD8⁺ T lymphocytes and NK cells, and induced robust immune memory. This synergistic engagement of the innate‐adaptive immune axis underscores its potential to address challenges in both local tumor control and systemic recurrence prevention.

## Graphical abstract

Preparation of a biopolymer‐based immune implant (BI) for the sustained release of R848 and oxaliplatin (OXA), enabling immune activation and preventing recurrence after radiofrequency ablation (RFA) treatment in hepatocellular carcinoma (HCC).

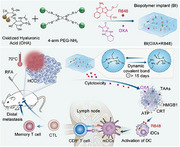

## 1 Introduction

Hepatocellular carcinoma (HCC), the predominant type of primary liver cancer, was reported as the sixth most commonly diagnosed malignancy and the third leading cause of cancer‐related mortality worldwide in 2022, underscoring its major impact on global health.^[^ ^1^, ^2^ ^]^ HCC alone accounts for ≈90% of all liver cancer cases, representing the dominant histological subtype.^[^ ^3^ ^]^ Alarmingly, ≈70% to 80% of patients are diagnosed at an advanced stage, limiting curative treatment options,^[^ ^4^, ^5^ ^]^ often with intrahepatic or extrahepatic metastases.^[^ ^6^, ^7^ ^]^ Due to the late stage at which hepatocellular carcinoma (HCC) is commonly diagnosed, only a small proportion of patients are eligible for curative strategies such as liver transplantation or surgical resection. As a result, long‐term survival remains markedly limited.^[^ ^8^, ^9^, ^10^ ^]^For multifocal and oligometastatic HCC, although non‐surgical systemic therapies, including immunotherapy and targeted therapy,^[^ ^11^, ^12^, ^13^ ^]^ have been approved for long‐term administration, they only control tumor growth and prevent tumor progression. Given the limitations of current treatments, exploring novel approaches for HCC management has become increasingly critical.

As a thermal ablation technique, RFA delivers high‐frequency alternating current in a minimally invasive manner to generate heat (typically 60–100 °C), resulting in coagulative necrosis of tumor tissues.^[^ ^14^, ^15^ ^]^ This technique is associated with minimal trauma, favorable tolerability, and a good safety profile.^[^ ^16^ ^]^ However, RFA is hindered by limitations such as the heat sink and carbonization effects, which not only result in incomplete ablation but also contribute to tumor recurrence and suboptimal long‐term outcomes by allowing residual viable tumor cells to persist at the ablation margins, particularly near large blood vessels where thermal injury is insufficient.^[^ ^17^, ^18^, ^19^, ^20^ ^]^ These deficiencies severely restrict the clinical utility of RFA in HCC treatment.

Recent studies indicate that necrotic tumor cells generated by local RFA may serve as a reservoir of tumor antigens, potentially initiating tumor‐specific immune reactions.^[^ ^21^ ^]^ This suggests a promising opportunity to utilize RFA as an immunological primer to enhance systemic antitumor immunity.^[^ ^22^, ^23^ ^]^ However, RFA monotherapy generates only a modest immunostimulatory effect and demonstrates limited capacity to induce robust systemic antitumor immune responses.^[^ ^24^, ^25^ ^]^ Moreover, the immunosuppressive microenvironment created at the ablation site can inhibit normal immune cell activity, thereby promoting tumor recurrence and metastasis.^[^ ^10^, ^26^ ^]^ Directly enhancing tumor cell inhibition while indirectly strengthening immunological effects to further suppress tumor growth represents a promising strategy for inducing complete ablation and reducing the recurrence risk of RFA.

**Scheme 1:**
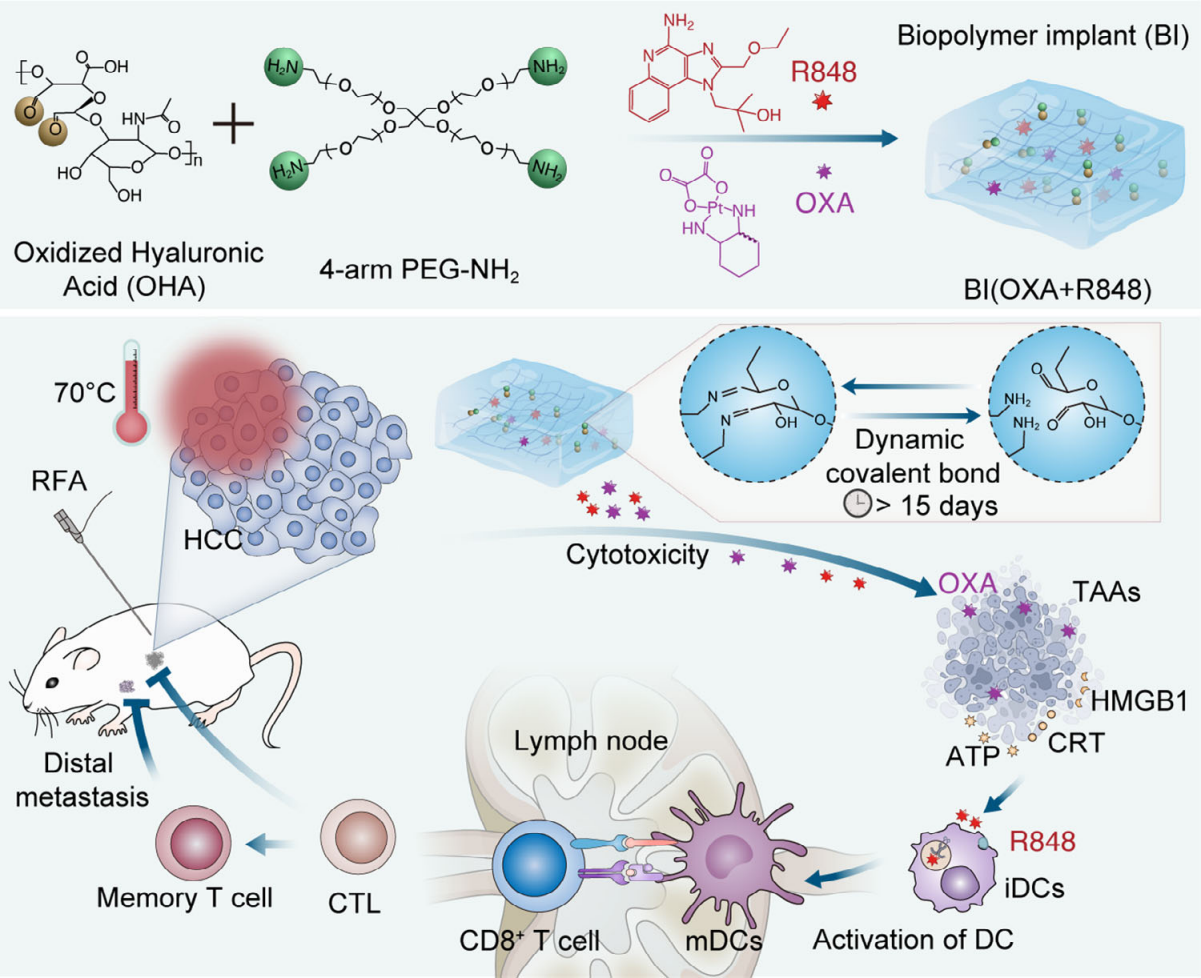
Schematic illustration of the preparation and mechanism of BI for post‐RFA hepatocellular carcinoma (HCC) therapy. The BI is crosslinked by oxidized hyaluronic acid (OHA) and 4‐arm PEG‐NH_2_. After radiofrequency ablation (RFA), the gel is minimally invasively injected into the tumor site, with the injection process assisted by laparoscopy and monitored in real‐time. Upon injection, both internal crosslinking within the hydrogel network and interface bonding between the aldehyde groups on the hydrogel and amine groups on tissue surfaces occur. The released oxaliplatin (OXA) works together with RFA to induce tumor cell death, promoting the release of tumor‐associated antigens. These antigens are captured by immature dendritic cells (DCs) and presented to naive T cells. In addition, the TLR7/8 agonist resiquimod (R848) is taken up by DCs, which activates them and enhances antigen presentation. This process promotes T cell infiltration into the tumor and generates immune memory, leading to more effective anti‐tumor immunity and death of HCC cells.

Herein, we report a novel, minimally invasive, and biopolymer‐based immune implant (BI) with in situ gelation and tissue‐adhesive properties, explicitly designed for localized immunotherapy following radiofrequency ablation (RFA) of hepatocellular carcinoma (HCC) (Scheme). Upon administration, the hydrogel rapidly undergoes gelation and adheres tightly to the ablation site, enabling sustained and localized drug release. In post‐RFA settings, we employed a BI hydrogel loaded with oxaliplatin (OXA) and resiquimod (R848) for site‐specific immunochemotherapy, demonstrating that this approach effectively prevents tumor recurrence in both subcutaneous and orthotopic liver tumor models in mice. This hydrogel platform provides a promising strategy to overcome the limitations of RFA and holds significant translational potential for future clinical application in HCC therapy (**Scheme** **1**).

## 2 Results and Discussion

### 2.1 Construction and Characterization of BI

**Figure 1:**
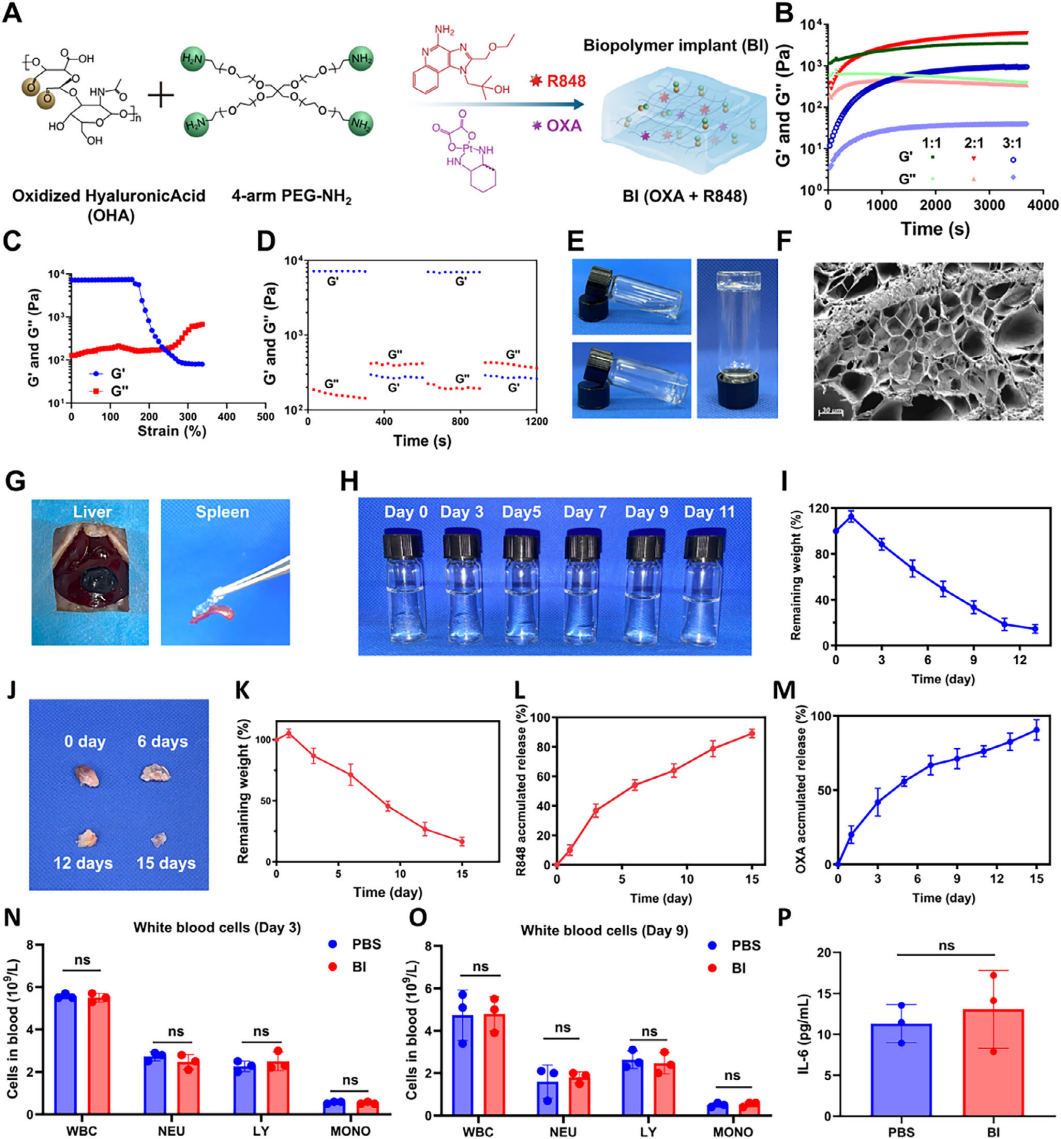
Construction and characterization of a biopolymer implant (BI). A) Schematic illustration of the construction process of the BI. B) Rheology properties of hydrogels formed with various 4‐arm PEG‐NH_2_/OHA mass ratios. C) Rheological analysis of the BI hydrogel under increasing strain, showing storage modulus (G′) and loss modulus (G″) as a function of strain (%). D) Rheological analysis of the hydrogel under alternating strain conditions, showing G′ and G″ over time during repeated strain cycles. E) Optical images of the hydrogel. F) SEM images of the hydrogel. G) Adhesion of the hydrogel to the liver and to the spleen in vitro. H) In vitro degradation of the hydrogel under physiological conditions. I) Degradation curve of the hydrogel in vitro (n = 3). J) Optical images of in vivo degradation experiments of implants. K) In vivo degradation curve of hydrogels (*n* = 3). L) Drug release curve of R848 (*n* = 3). M) Drug release curve of OXA (*n* = 3). The complete blood count after injection of PBS or BI on the stomach at day 3 (N) and day 9 (O) (*n* = 3). P) The IL‐6 concentration in the serum after injection of PBS or BI at day 3 (*n* = 3).

To meet the clinical demand for preventing tumor recurrence and residual growth following RFA, we developed a biopolymer implant system based on Schiff base crosslinking between OHA and 4‐arm PEG‐NH_2_ (**Figure** **1**A). To verify the structure and purity of individual components, we first conducted structural characterization. The structure of R848 was confirmed using ^1^H NMR in DMSO‐*d*_6._ The structure of OHA was confirmed using ^1^H NMR in DMSO‐*d*_6_ and ^13^C NMR in D_2_O, which displayed a characteristic resonance corresponding to the aldehyde group (Figure S2, Supporting Information). The actual oxidation degree of the aldehyde groups was 36%. FITR analysis revealed a characteristic IR peak of OHA at 1740 cm^−1^ (Figure S3, Supporting Information), consistent with the formation of aldehyde groups. Additionally, 4‐arm PEG‐NH_2_ was confirmed by ^1^H NMR in CDCl_3_ (Figure S4, Supporting Information), verifying the expected polymer structure. Additionally, the FTIR spectrum of the freeze‐dried hydrogel BI confirms the formation of imine (C═N) bonds in the hydrogel matrix (Figure S5, Supporting Information).

We next investigated the mechanical performance of the hydrogels formed with different mass ratios of 4‐arm PEG‐NH_2_ to OHA using a rheometer. As the mass ratio increased from 1:1 to 3:1, the storage modulus (G') initially increased and then decreased, reaching a peak value above 6000 Pa at a ratio of 2:1 (Figure 1B). Injectability was evaluated by shear‐thinning tests, which demonstrated a gradual decrease in storage modulus with increasing shear force (Figure 1C). Furthermore, alternating the shear strain between 1% and 500% confirmed the gel's self‐healing capability, as it reversibly switched its storage modulus from ≈6000 Pa to 400 Pa (Figure 1D). The gelation process was evaluated using the vial tilting method. Upon mixing the OHA and 4‐arm PEG‐NH_2_ solutions, gelation was observed at 2 min 5 s, 2 min 10 s, and 2 min 30 s in three independent trials. The average gelation time was 2 min 15 ± 12.7 s (n = 3). The gelation behavior was also confirmed using both tube inversion and tilting tests (Figure 1E, F). Scanning electron microscopy revealed a porous, interconnected internal network within the lyophilized hydrogel, with pore sizes ranging from 10 to 30 µm (Figure 1F). Given that appropriate adhesion strength is important to ensure stable adhesion of the hydrogel to soft tissue surfaces such as the liver or tumor periphery following RFA, the adhesion strength of BI was assessed. As depicted in Figure S6 (Supporting Information), the BI hydrogel achieved an average adhesion strength of 21.19 ± 0.02 kPa. As expected, the hydrogel showed strong adhesion to biological tissues, including liver and spleen (Figure 1G). In vitro degradation analysis revealed an initial swelling phase, followed by a gradual mass loss, with the gel retaining only 18.5% of its original weight after 11 days in PBS (pH 7.4) at 37 °C(Figure 1H, I). In vivo studies confirmed that the BI hydrogel remained at the subcutaneous site for over 15 days (Figure 1J, K). The loading drug doses of OXA and R848 were selected based on our previous literature.^[^ ^27^, ^28^ ^]^ In addition, we conducted in vitro cytotoxicity assays to determine the IC50 values of OXA and R848 against H22 hepatocellular carcinoma cells. The IC50 values were determined to be 90.48 µM for OXA and 160.0 µM for R848, respectively (Figure S7, Supporting Information). Controlled drug release studies showed that R848 was released in a sustained manner, with cumulative release exceeding 85% within 14 days (Figure 1L). In addition, OXA exhibited a similar release pattern, with 90.6 ± 6.9% of the drug released within two weeks (Figure 1M). To evaluate whether enzymatic degradation in the tumor microenvironment might accelerate drug release, we conducted additional studies in the presence of hyaluronidase (HAase, 5 mg mL^−1^). While a slight increase in initial drug release was observed, the differences did not reach statistical significance (*P* > 0.05, Figure S8, Supporting Information), indicating that the hydrogel has not yet been found to be enzyme‐responsive.To assess the systemic biocompatibility of BI, blood cell counts were measured on days 3 and 9 post‐injection. No significant changes were observed in white blood cells (WBC), neutrophil (NEU), lymphocyte (LY), or monocyte (MONO) levels (Figure 1N, O). Additionally, serum IL‐6 concentrations remained stable, indicating the absence of systemic inflammation (Figure 1P). For in vitro cytocompatibility testing, we conducted MTT assays by incubating H22, NIH/3T3, and HCAEC cells with the hydrogel for 48 h. As shown in Figure S9 (Supporting Information), the viability of 3T3 and HCAEC cells remained above 80%, supporting the biosafety of the material.

These findings demonstrated that the BI hydrogel exhibited excellent mechanical strength, controlled biodegradability, sustained drug release, and high biocompatibility, indicating its potential as an effective adjunct to RFA for suppressing tumor growth and preventing recurrence.

### 2.2 RFA + BI (OXA + R848) for Treatment of subcutaneous HCC Model

**Figure 2:**
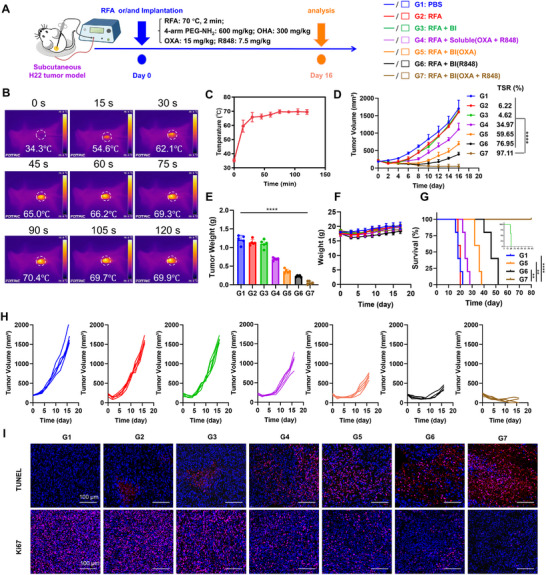
Synergistic treatment of subcutaneous hepatocellular carcinoma using radiofrequency ablation combined with a BI. A) Image of the equipment setup for radiofrequency ablation and schematic illustration of the therapeutic strategy used in the subcutaneous HCC model. B) Infrared thermal images showing the temperature evolution at the tumor site during RFA treatment; the final temperature reached 70 °C and was maintained for 2 min. C) Quantification of tumor surface temperature over time during RFA (*n* = 3). D) Tumor growth curves following different treatments. (*n* = 5). E) Tumor weights measured at the experimental endpoint across therapeutic groups. (*n* = 5). F) Body weight of mice during treatment in each group (*n* = 5). G) Survival curves of mice receiving different therapeutic regimens(*n* = 5). H) Individual tumor growth trajectories of H22‐bearing mice under each treatment condition (*n* = 5). I) Immunohistochemical staining of tumor tissues with Ki67 and TUNEL to assess proliferation and apoptosis post‐treatment. Scale bar = 100 µm.

To investigate the anti‐tumor effect of BI hydrogel after RFA, an H22 tumor‐bearing mouse model was established, and a standardized RFA protocol was applied (**Figure** **2**A). In subcutaneous HCC model, tumors were ablated for 120 s using RFA once their volumes reached ≈150 mm^3^. Infrared thermography (IR) was used to monitor the ablation process, providing real‐time heat distribution at the tumor site (Figure 2B), with surface temperatures reaching 70 °C within 2 min (Figure 2C). Mice were randomly divided into seven groups: G1, untreated control; G2, RFA alone; G3, RFA + blank BI hydrogel; G4, RFA + soluble R848 and OXA; G5, RFA + BI hydrogel loaded with OXA; G6, RFA + BI hydrogel loaded with R848; and G7, RFA + BI hydrogel co‐loaded with R848 and OXA. Tumor volume measurements (Figure 2D) showed that RFA alone (G2) exerted limited antitumor effects, whereas the combination of RFA with BI(R848 + OXA) (G7) significantly suppressed tumor growth. Tumor volume measurements (Figure 2D) revealed that RFA alone (G2) resulted in minimal tumor suppression (TSR: 6.22%), with rapid tumor regrowth observed. The addition of soluble R848 and OXA post‐RFA (G4) significantly enhanced antitumor efficacy (TSR: 34.97%), highlighting the benefit of immunochemotherapy. Notably, the combination of RFA with BI hydrogel co‐loaded with R848 and OXA (G7) achieved the most pronounced and sustained tumor inhibition (TSR: 97.11%), attributed to the prolonged local release of therapeutic agents from the BI hydrogel. Consistently, tumor weights measured on day 16 (Figure 2E) confirmed that G7 achieved the most significant reduction in tumor burden among all groups (*P* < 0.05). To assess systemic side effects, body weights were monitored throughout the treatment period (Figure 2F). Mice receiving soluble R848 + OXA (G4) experienced notable weight loss by day 3, likely due to acute systemic toxicity from rapid drug diffusion. In contrast, the G7 group maintained stable body weight, suggesting that the hydrogel's localized and sustained release mitigated systemic exposure. Survival outcomes further reinforced the therapeutic benefits of the hydrogel‐based delivery system (Figure 2G). While mice in the RFA‐only group (G2) exhibited a median survival of 20 days, those in G7 demonstrated significantly prolonged survival, with two mice achieving complete tumor remission. To better characterize treatment responses, individual tumor growth curves were analyzed (Figure 2H), revealing that only G7 achieved durable and near‐complete tumor suppression. In contrast, the soluble drug group (G4) showed moderate tumor inhibition and a limited survival benefit. To investigate the underlying mechanisms at the cellular level, immunohistochemical analysis of tumor sections was performed using Ki67 and TUNEL staining (Figure 2I). The RFA + BI(R848 + OXA) group exhibited markedly reduced proliferation and elevated apoptosis compared to the other groups, indicating that the hydrogel‐based combination therapy not only suppressed tumor growth but also effectively induced tumor cell death. In addition to therapeutic efficacy, we further evaluated the biosafety of the combination treatment. H&E staining of major organs, including liver, kidney, heart, lung, and spleen, revealed no significant histopathological abnormalities across all groups (Figure S10, Supporting Information), suggesting minimal off‐target tissue damage. Furthermore, serum biochemical parameters, including ALT, AST, BUN, and AKP, were measured in a parallel H22 tumor model to assess liver and kidney function (Figure S11, Supporting Information). The RFA + BI(R848 + OXA) group exhibited no significant elevations in these markers compared to the PBS group, further confirming the systemic safety of the hydrogel‐based delivery system. Together, these findings demonstrate that the BI hydrogel plays a critical role in the co‐delivery of R848 and OXA in combination with RFA, enabling sustained local drug release and enhanced immune activation. This hydrogel‐based strategy achieved synergistic tumor control while minimizing systemic toxicity.

### 2.3 Regulation of Tumor Immune Microenvironment and Immune Memory by RFA Combined with BI (OXA + R848)

Effective antitumor immunity relies on coordinating the interactions between innate and adaptive immune responses. To elucidate the immunomodulatory effects of RFA combined with BI(R848 + OXA), flow cytometric analysis of tumor‐infiltrating immune cells was conducted in HCC‐bearing mice.

**Figure 3:**
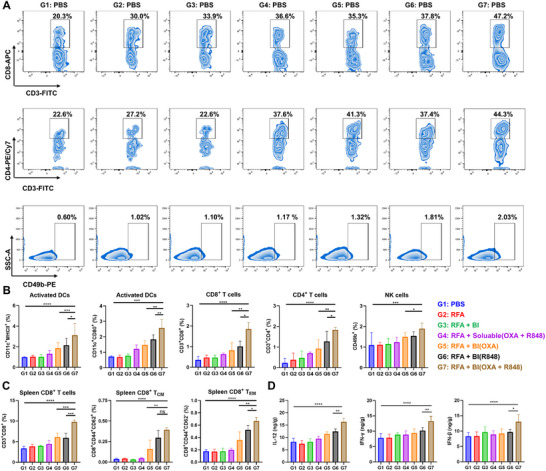
Regulation of Antitumor Immunity by RFA Combined with BI(R848 + OXA). A) Representative flow cytometry dot plots showing tumor‐infiltrating CD8^+^ T cells, CD4^+^ T cells, and NK cells following different treatments. B) Quantification of activated dendritic cells (CD11c^+^CD80^+^ and CD11c^+^MHCII^+^), CD8^+^ T cells, CD4^+^ T cells, and NK cells in tumors (*n* = 5). C) Flow cytometric analysis of splenic CD8^+^ T cells, including central memory (T_CM_) and effector memory (T_EM_) subsets (*n* = 5). D) ELISA quantification of IL‐12, IFN‐γ, and IFN‐β levels in tumor tissues (*n* = 5).

Natural killer (NK) cells, as critical effectors of the innate immune system, provide early defense by directly lysing tumor cells and secreting cytokines that shape subsequent adaptive responses.^[^ ^29^, ^30^, ^31^, ^32^ ^]^ In the RFA + BI(R848 + OXA) group (G7), tumor‐infiltrating NK cells were increased by 1.52, 1.26, and 1.23 fold relative to G4, G5, and G6, respectively (**Figure** 3A‐B). These results indicate that the combination therapy enhanced innate immune surveillance and cytotoxic potential at the tumor site. Dendritic cells (DCs) serve as a bridge between innate sensing and adaptive activation through antigen uptake and presentation. The G7 group exhibited significantly increased proportions of activated DCs.^[^ ^33^, ^34^, ^35^ ^]^ CD11c⁺MHCII⁺ DCs were increased by 2.38, 1.68, and 1.45fold, and CD11c⁺CD80⁺ DCs were increased by 2.12, 1.75, and 1.40 fold, when compared with G4, G5, and G6, respectively (Figure 3B; gating strategy in Figure S12, Supporting Information). These data indicate that the hydrogel‐based treatment augmented antigen presentation capacity and co‐stimulatory signaling. Additionally, adaptive immune activation is crucial for the eradication of residual tumor cells and the establishment of sustained immune surveillance. Among adaptive immune effectors, CD8⁺ cytotoxic T lymphocytes (CTLs) are primarily responsible for directly killing tumor cells through the release of perforin and granzyme. In contrast, CD4⁺ helper T cells support antitumor immunity by enhancing antigen presentation, promoting CTL expansion, and aiding in the generation of memory responses.^[^ ^36^, ^37^, ^38^ ^]^ Flow cytometric analysis revealed that combination treatment with RFA + BI(R848 + OXA) markedly enhanced T cell responses in the tumor microenvironment. Specifically, CD8⁺ T cell infiltration was increased by 2.94, 2.26, and 1.83 fold, and CD4^+^ T cells by 2.63, 2.00, and 1.45 fold, compared to the G4, G5, and G6 groups, respectively (Figure 3B; gating strategy in Figure S13, Supporting Information). These results indicate that the combination therapy robustly enhanced both cytotoxic and helper T cell recruitment and activation at the tumor site, likely through improved antigen presentation and immune stimulation.

To assess whether the observed T cell activation was associated with durable immune protection, CD8^+^ memory T cell subsets in the spleen were analyzed. Central memory T cells (T_CM_, CD44^+^CD62L^+^) and effector memory T cells (T_EM_, CD44^+^CD62L^−^) are essential for long‐term immune surveillance and rapid secondary responses to tumor‐associated antigens. Compared with the PBS group (3.37 ± 0.44%), the G7 group showed a significantly elevated proportion of splenic CD8⁺ T cells (9.79 ± 0.40%) (Figure 3C). Notably, while G5 and G6 induced moderate increases in T_CM_ and T_EM_ subsets, the G7 group exhibited the most pronounced memory response, with T_CM_ reaching 0.39 ± 0.02% and T_EM_ 0.67 ± 0.05% (gating strategy in Figure S14, Supporting Information). This memory profile suggests a strong potential for long‐term tumor control and recurrence prevention. The levels of key proinflammatory cytokines in the tumor were assessed using ELISA. IL‐12 is a central cytokine in orchestrating innate‐adaptive crosstalk by promoting NK cell cytotoxicity and enhancing Th1 differentiation.^[^ ^39^ ^]^ IFN‐γ, primarily produced by activated T cells and NK cells, is crucial for macrophage activation, MHC upregulation, and direct inhibition of tumor cells.^[^ ^40^, ^41^ ^]^ IFN‐β, a type I interferon, also contributes to antigen presentation and antiviral‐like antitumor immunity.^[^ ^42^ ^]^ The RFA + BI (R848 + OXA) group showed the highest expression of all three cytokines (Figure 3D), indicating that the combination therapy achieved immune activation

In summary, the results demonstrated that the combination of RFA and BI(R848 + OXA) effectively modulated the tumor immune microenvironment by enhancing innate effector cell activity, promoting antigen presentation, expanding cytotoxic and helper T cells, inducing durable memory responses, and elevating cytokine‐mediated immune signaling. This comprehensive immune response contributes to the strong and lasting antitumor effects observed in vivo and highlights the potential of this approach for HCC treatment.

### 2.4 RNA‐Seq Analysis for Treatment of Combination Therapy

**Figure 4:**
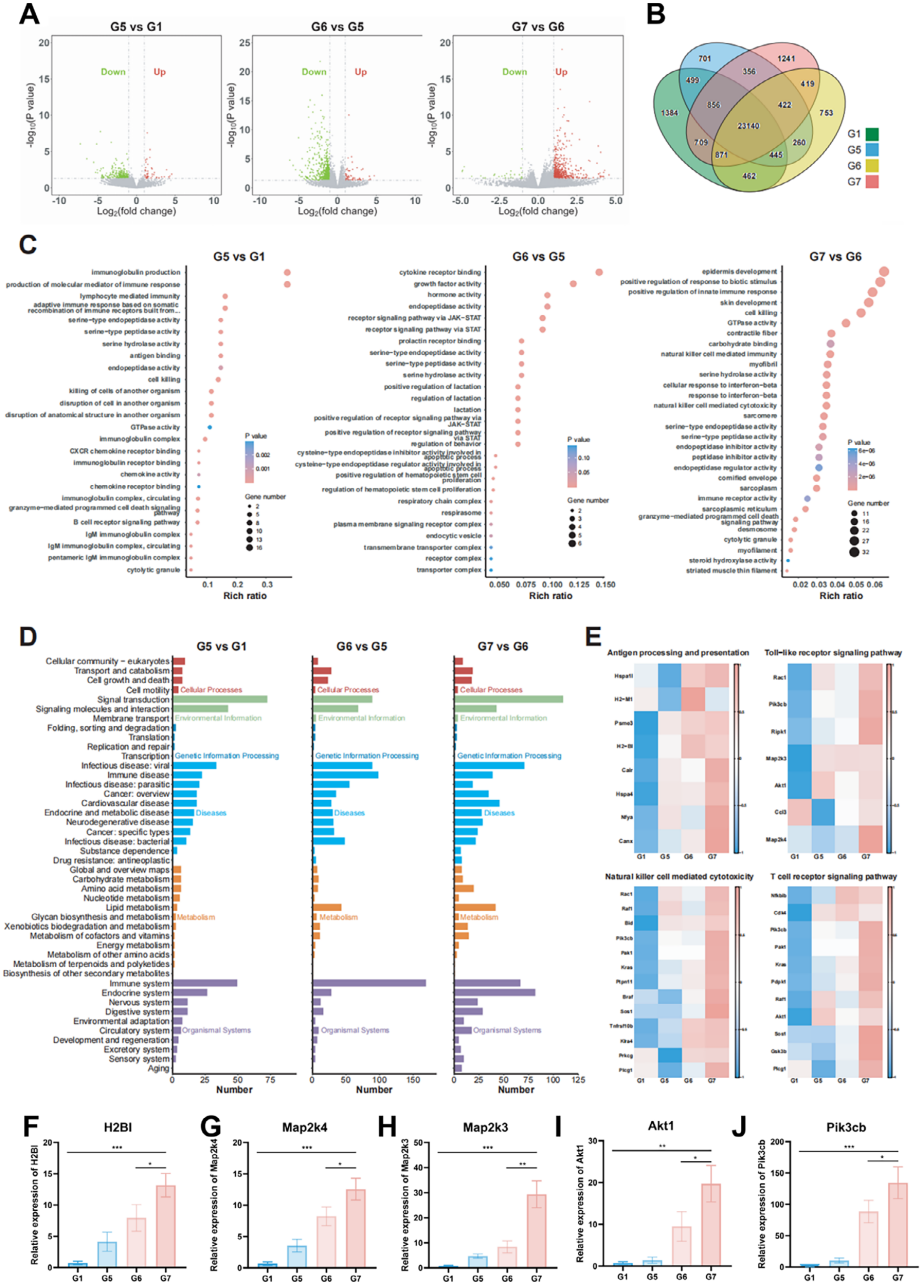
Transcriptomic Profiling and Validation of Immune‐Related Pathways in HCC Tumors Following Treatment. A) Volcano plots comparing gene expression profiles between untreated control (G1) and each treatment group, with significantly upregulated (red) and downregulated (green) genes highlighted. B) Venn diagram showing overlaps in differentially expressed genes (DEGs) among four groups: control (G1), RFA + BI(OXA) (G2), RFA + BI(R848) (G3), and RFA + BI(OXA + R848) (G4). C) KEGG pathway enrichment analysis of DEGs across treatment comparisons. D) Functional clustering of DEGs involved in key biological processes based on KEGG annotations. E) Heatmaps showing representative genes enriched in four immune‐related pathways: antigen processing and presentation, Toll‐like receptor signaling, NK cell‐mediated cytotoxicity, and T cell receptor signaling. (F‐J) RT‐qPCR validation of selected DEGs, including H2‐BI (F), Map2k4 (G), Map2k3 (H), Akt1 (I), and Pik3cb (J), showing relative mRNA expression levels between different groups. All data represent mean ± SD from biological triplicates (*n* = 3).

To further investigate the molecular mechanisms underlying the enhanced therapeutic efficacy of RFA + BI(R848 + OXA), we performed whole‐transcriptome RNA sequencing (RNA‐seq) on tumors collected from treated HCC‐bearing mice. As shown in **Figure** **4**A, volcano plot analysis revealed differentially expressed gene (DEG) profiles across treatment groups. Compared to the PBS group (G1), the RFA + BI(OXA) group (G5) exhibited 366 differentially expressed genes (DEGs), including 47 upregulated and 319 downregulated genes (*P* < 0.05, fold change ≥ 2). Relative to G5, the RFA + BI(R848) group (G6) displayed 657 DEGs (68 upregulated, 589 downregulated). Notably, the RFA + BI(R848 + OXA) group (G7) induced a markedly different expression profile, with 564 genes upregulated and only 28 downregulated compared to G6. These findings indicate that the sustained co‐delivery of R848 and OXA via the BI hydrogel substantially altered the transcriptional landscape, highlighting the immunomodulatory potential of the localized delivery system. To compare the DEG distributions among the four groups and to support downstream pathway analysis, we constructed a Venn diagram (Figure 4B). This allowed visualization of the distinct gene sets regulated by each treatment, serving as a reference for subsequent functional annotation and enrichment analysis.

KEGG pathway enrichment revealed treatment‐specific immunological signatures (Figure 4C). In the RFA + BI(OXA) group (G5), DEGs were predominantly associated with cell killing, consistent with oxaliplatin's known cytotoxic effects and suggesting enhanced tumor cell death facilitated by local retention and release from the hydrogel. In the RFA + BI(R848) group (G6), pathway enrichment indicated R848‐induced immune activation, with significant involvement of the receptor signaling pathway via JAK‐STAT and its positive regulation. These are canonical downstream targets of TLR7/8 signaling and are indicative of MyD88‐mediated activation of type I interferon responses. Additional pathways related to hematopoietic cell regulation and antigen transport were also upregulated, which is consistent with innate immune priming. In the RFA + BI(R848 + OXA) group (G7), a broader immunological shift was observed. Enriched pathways included positive regulation of innate immune response, cell killing, natural killer (NK) cell‐mediated immunity, immune receptor activity, and the granzyme‐mediated programmed cell death signaling pathway. These findings indicate the combined impact of OXA‐induced immunogenic cell death and R848‐mediated immune stimulation, resulting in coordinated activation of both innate and adaptive immune responses. Further functional classification of DEGs (Figure 4D) demonstrated significant involvement in pathways related to immune regulation, signal transduction, metabolic remodeling, and tissue homeostasis. These results suggest that hydrogel‐mediated sustained delivery not only promotes immune activation but also modulates tumor‐intrinsic signaling and the surrounding microenvironment, contributing to the observed therapeutic benefit.

To further investigate immune‐related transcriptional changes, we performed heatmap analysis of genes involved in key immune pathways (Figure 4E). Compared to all other groups, the RFA + BI(R848 + OXA) group (G7) showed notably increased expression of genes associated with antigen processing and presentation, Toll‐like receptor signaling, NK cell‐mediated cytotoxicity, and T cell receptor signaling. These transcriptional patterns were consistent with earlier flow cytometry results, which showed enhanced infiltration and activation of CD8^+^ and CD4^+^ T cells, NK cells, and dendritic cells, indicating broad immune activation.

To validate these results and clarify the effect on adaptive immunity, we selected six representative genes, including H2‐BI, Rac1, Map2k4, Map2k3, Akt1, and Pik3cb, for RT‐qPCR analysis (Figure 4F–J). Each gene was chosen based on its functional involvement in the pathways identified above. H2‐BI, a gene critical for MHC class I‐mediated antigen presentation, was significantly upregulated in the G7 group compared with G1, G5, and G6, suggesting improved antigen availability for CD8^+^ T cell priming (Figure 4F). Map_2k4 a_nd Map2k3, key components of the Toll‐like receptor (TLR) signaling pathway, were also highly expressed in G7 (Figure 4G, H). These genes are involved in MAPK cascade activation and downstream transcription of inflammatory mediators. Their upregulation indicates enhanced innate immune sensing that may support T cell activation via dendritic cell maturation and cytokine production. Akt1 and Pik3cb are associated with T cell receptor signaling and NK cell‐mediated cytotoxicity. In G7, both genes were significantly upregulated relative to G1, G5, and G6, suggesting stronger lymphocyte activation and effector function (Figure 4I). In particular, Pik3cb is involved in downstream PI3K‐Akt signaling essential for T cell survival and proliferation, as well as NK cell cytotoxicity (Figure 4J). Taken together, the RT‐qPCR results confirm that the RFA + BI(R848 + OXA) group shows the highest expression of genes involved in antigen presentation, TLR signaling, and cytotoxic lymphocyte activation. These findings support the conclusion that RFA and sustained co‐delivery of R848 and OXA via BI could enhance transcriptional programs underlying adaptive immune responses, especially through improved T cell priming, activation, and effector activity.

These molecular signatures provide further mechanistic evidence that the combination therapy promotes sustained antitumor immunity, providing a molecular explanation for the enhanced immune cell infiltration and cytokine production observed in previous experiments.

### 2.5 Systemic Anti‐tumor Efficacy of BI in Rechallenge and Bilateral HCC Tumor Models

**Figure 5:**
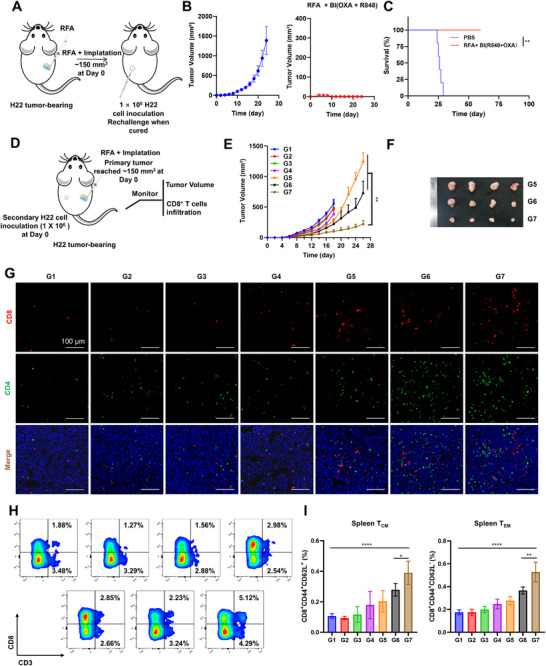
Systemic Anti‐tumor Efficacy of BI Hydrogel in Rechallenge and Bilateral HCC Tumor Models. A) Schematic diagram of the experimental design for the HCC tumor rechallenge model (*n* = 5). B) Tumor growth curves in rechallenged mice, comparing previously cured RFA + BI(R848 + OXA)‐treated mice with naive controls. C) Survival curves of mice following different treatments (*n* = 5). D) Schematic of bilateral HCC tumor inoculation and treatment strategy. E) Tumor volume changes in distant (non‐ablated) tumors after various treatment regimens (*n* = 4). F) Representative images of distant tumors collected on day 16 post‐treatment (*n* = 4). G) Confocal images showing CD4^+^ and CD8^+^ T cell infiltration in distant tumors on day 16. H) Representative immunofluorescence images of memory CD8^+^ T cells in spleens on day 16. I) Flow cytometric analysis of splenic central memory (T_CM_) and effector memory (T_EM_) CD8^+^ T cells across treatment groups (*n* = 4).

To evaluate the durability of immune protection induced by combination therapy, a tumor rechallenge experiment was performed as outlined in **Figure** **5**A. Thirty days after complete tumor regression, mice previously cured with RFA + BI(R848 + OXA) were reinoculated with H22 tumor cells on the contralateral flank (left lateral abdominal region). As shown in Figure 5B, all rechallenged mice in the treatment group exhibited complete protection against tumor growth, with no detectable tumors throughout the 30‐day observation period, while naive control mice developed rapidly growing tumors. These findings indicate that the combination therapy elicited a potent and durable immunological memory capable of completely preventing tumor recurrence. Survival analysis further confirmed this effect, with all mice in the treatment group surviving beyond 60 days, whereas naive mice succumbed within 30 days (Figure 5C). To further investigate systemic immune responses, a bilateral HCC tumor model was established, and only the primary tumor received RFA and hydrogel treatment (Figure 5D). Tumor volume measurements revealed that both the RFA + BI(OXA) and RFA + BI(R848) groups significantly suppressed the growth of distant tumors compared to the control groups. Notably, the RFA + BI(R848 + OXA) group exhibited the most significant inhibitory effect on the untreated tumors (Figure 5E). Representative images of tumors from endpoint‐collected mice show substantial tumor reduction in the combination group (Figure 5F). Immunological analysis of distant tumors at day 16 revealed pronounced infiltration of CD4^+^ and CD8^+^ T cells in the RFA + BI(R848 + OXA) group, as shown by confocal imaging (Figure 5G), suggesting effective activation and trafficking of adaptive immune effector cells. Concurrently, spleens collected on day 16 from the same cohort showed increased numbers of CD8^+^ memory T cells (Figure 5H), and flow cytometry analysis confirmed that both central memory (T_CM_; CD44^+^CD62L^+^) and effector memory (T_EM_; CD4^+^CD62L^−^) CD8^+^ T cell populations were significantly elevated compared to other treatment groups (Figure 5I). These findings indicate that, through the hydrogel's ability to provide sustained intratumoral release of both agents, the combination therapy not only elicited a robust immune response but also promoted the generation of long‐term immunological memory, contributing to both immediate tumor suppression and durable recurrence prevention.

### 2.6 Therapeutic Efficacy of BI Hydrogel in an Orthotopic Liver Cancer Model

**Figure 6:**
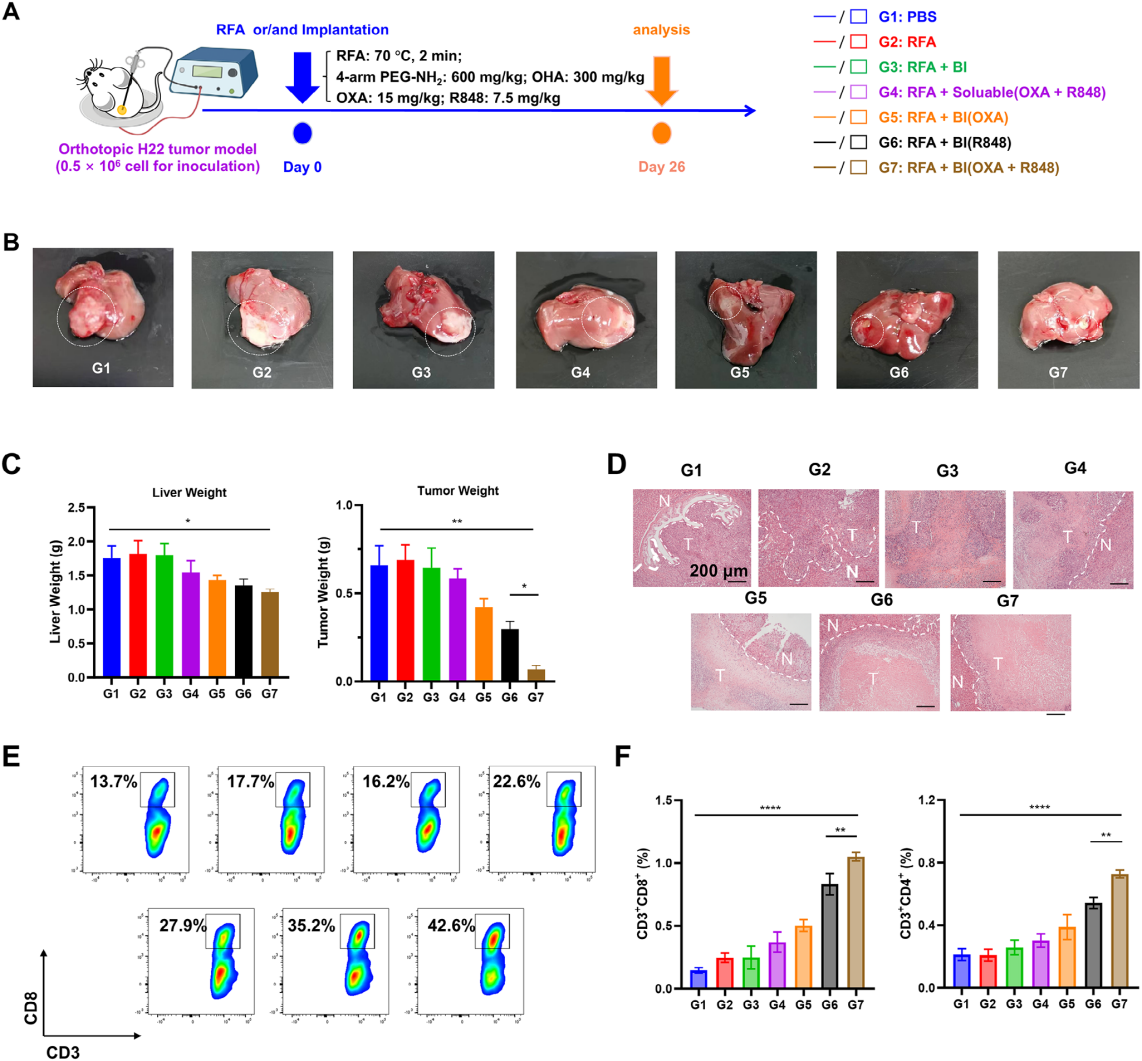
Therapeutic Efficacy of BI Hydrogel in an Orthotopic Liver Cancer Model. A) Schematic diagram showing the procedure for orthotopic HCC tumor inoculation and treatment allocation. B) Representative liver images collected after treatment illustrating differences in tumor burden across groups. C) Quantification of liver weight and tumor weight in each treatment group (*n* = 4). D) H&E staining of liver and tumor tissues after different treatments, showing histopathological features (Scale bar = 200 µm). E) Representative flow cytometry plots of intratumoral CD8^+^ T cell infiltration in orthotopic tumors. F) Quantitative analysis of CD4^+^ and CD8^+^ T cell infiltration in orthotopic liver tumors by flow cytometry (*n* = 4).

To evaluate the therapeutic potential of the BI hydrogel in a clinically relevant context, we established an orthotopic HCC model by injecting 5 × 10^5^ H22 cells directly into the left hepatic lobe of BALB/c mice, thereby simulating intrahepatic tumor growth. Seven days after tumor cell inoculation, mice were randomly divided into seven treatment groups (**Figure** **6**A): (1) Control (G1), (2) RFA (G2), (3) RFA + BI (G3), (4) RFA + Soluble (OXA + R848) (G4), (5) RFA + BI(OXA) (G5), (6) RFA + BI(R848) (G6), and (7) RFA + BI(OXA + R848) (G7). Gross examination of the liver specimens post‐treatment revealed substantial tumor burden in the control, RFA, and RFA + BI groups, whereas the RFA + BI(OXA + R848) group showed markedly reduced tumor mass (Figure 6B). Quantitative analysis of liver and tumor weights further supported these observations, with the combination group demonstrating the greatest reduction in tumor burden (Figure 6C).

Histopathological analysis of liver tissues by H&E staining provided further evidence of therapeutic efficacy. The control group exhibited dense tumor infiltration with uniform, viable nuclei, while the RFA + BI(OXA + R848) group showed extensive areas of necrosis and disrupted tumor architecture, indicating significant tumor cell death induced by the combination therapy (Figure 6D). To evaluate immune activation, flow cytometry was performed on tumor‐infiltrating lymphocytes. Representative plots demonstrated elevated CD8^+^ T cell presence in tumors from the combination group (Figure 6E). Quantitative analysis confirmed that both CD4^+^ and CD8^+^ T cell infiltration were significantly higher in the RFA + BI(OXA + R848) group compared to other treatment arms (Figure 6F), suggesting that the enhanced therapeutic efficacy is associated with robust T cell‐mediated immune responses within the tumor microenvironment.

These findings confirmed that RFA combined with BI(R848 + OXA) not only inhibits orthotopic liver tumor growth but also facilitates effective immune activation, offering a promising strategy for treating HCC.

## 3 Conclusion

In this study, we developed a BI hydrogel loaded with OXA and R848 for post‐RFA liver cancer immunotherapy. The BI hydrogel demonstrated rapid in situ gelation and strong tissue adhesion, enabling sustained local drug release. In vivo studies showed that RFA + BI (OXA + R848) achieved potent tumor inhibition in subcutaneous models and significantly prolonged survival in orthotopic liver cancer models. Moreover, this combination therapy effectively suppressed both primary and distant tumors in a bilateral model, indicating the induction of systemic anti‐tumor immunity. Mechanistic investigations revealed enhanced dendritic cell activation, increased intratumoral infiltration of CD8^+^ T cells and NK cells, and the establishment of durable immune memory. These results suggest that the BI hydrogel not only improves local tumor control but also elicits a robust immune response. Accordingly, the BI hydrogel system is anticipated to have significant translational potential in HCC immunotherapy.

## 4 Experimental Section

### Materials

Resiquimod (R848) was purchased from Sigma–Aldrich (St. Louis, MO, USA). Hyaluronic acid was obtained from Macklin Biochemical Co., Ltd. (Shanghai, China), and 4‐arm poly(ethylene glycol) amine (4‐arm PEG‐NH_2_, 10 kDa) was purchased from JINGPI TECHNOLOGY (Beijing, China). Hyaluronidase, deoxyribonuclease I (DNase), and collagenase were supplied by Source Leaf (Beijing, China). Dulbecco's phosphate‐buffered saline (PBS), fetal bovine serum (FBS), and penicillin‐streptomycin were all obtained from Gibco (Thermo Fisher Scientific, USA). Mycoplasma testing kits (Beyotime, China) were used to ensure all cell cultures were free of contamination. Scanning electron microscopy (SEM) was conducted using a JSM‐7000F system (JEOL Ltd, Tokyo, Japan). Rheological measurements were carried out on a Physica MCR 301 rheometer (Anton Paar). For radiofrequency ablation (RFA), a MEDSPHERE S‐500 RF generator was used.

### Synthesis of Oxidized Hyaluronic Acid (OHA)

Hyaluronic acid (4.0 g) was dissolved in distilled water under stirring at room temperature. Upon complete dissolution, sodium periodate (900.0 mg) was added, and the reaction was maintained for 24 h. The resulting mixture was purified by dialysis in deionized water for 3 days (MWCO 3500 Da) and lyophilized to yield oxidized hyaluronic acid (OHA). Structural characterization was performed using both ^1^H and ^13^C NMR spectroscopy (Bruker AV‐500).

### The Construction of BI

Hydrogels were formed via Schiff‐base cross‐linking of 4‐arm PEG‐NH_2_ and OHA at a total polymer concentration of 10% (w/v). Specifically, 10 wt% solutions of PEG‐NH_2_ and OHA were prepared in water. 300 µg OXA was directly dissolved into the PEG‐NH_2_ solution, followed by the addition of R848 (150 µg in 10 µL DMSO), corresponding to ≈1064 µg mL^−1^ of OXA and 532 µg mL^−1^ of R848, respectively. Equal volumes of PEG‐NH_2_ and OHA solutions were then combined, vortexed for 15 s, and 300 µL of the mixture was transferred into 48‐well culture plates.

### Safety Evaluation of the BI

To evaluate the biocompatibility of blank gels (BI), female BALB/c mice were randomly assigned to two groups: PBS and BI (gels implanted). Peripheral blood was collected on days 3 and 9 after treatment for hematological analysis using a MEK‐6318 analyzer, including leukocyte subtyping and quantification. Serum IL‐6 levels were also measured to assess systemic inflammatory responses.

### Rheological Experiments

Rheological characterization was conducted using a Physica MCR 301 rheometer (Anton Paar) equipped with a 25 mm parallel‐plate geometry. Copolymer solutions were loaded at 37 °C with a fixed gap of 0.4 mm. To ensure measurements remained within the linear viscoelastic region, oscillatory shear tests were performed at a constant frequency of 1 Hz and strain of 1%. The evolution of the storage modulus (G') and loss modulus (G″) over time was recorded to monitor gelation kinetics. To minimize solvent evaporation during measurement, the sample perimeter was sealed with a thin layer of silicone oil.

For the shear‐thinning experiments, the shear force was gradually increased from 1% to 500%, and changes in the gel's storage modulus and loss modulus were observed. In contrast, for the self‐healing performance tests, the shear force was rapidly alternated between 1% and 500%, cycling three times. The corresponding changes in the storage modulus and loss modulus of the gel were recorded during this process.

### In Vitro and In Vivo Degradation Analyses

For degradation experiments, hydrogels were assessed both in vitro and in vivo. For in vivo degradation, hydrogels were implanted subcutaneously near the tumors in BALB/c mice. At predetermined time points (6, 12, 15 days), the mice were euthanized, and the tumors, along with the remaining hydrogels, were excised. Photographs were taken to document the degradation process, and the hydrogels were carefully separated from the surrounding tissue, rinsed with PBS, dried, and weighed. The residual weight was used to calculate the percentage of degradation over time. For in vitro degradation, hydrogels were placed in transparent vials containing PBS and incubated at 37 °C with constant shaking (90 rpm). At predefined intervals, the hydrogels were removed, dried, and weighed to assess their degradation. Both degradation studies were conducted in triplicate, with results presented as mean ± SD.

### In Vitro Release of R848 and OXA From Implants

To evaluate the pH‐responsive release characteristics of R848, hydrogel implants loaded with the drug were incubated under simulated physiological (pH 7.4). Each sample was immersed in 2 mL PBS and maintained at 37 °C in a shaking incubator (90 rpm) to mimic in vivo dynamic environments. At predetermined time intervals, the supernatant was replaced with fresh buffer to maintain sink conditions. The released R848 was quantified by high‐performance liquid chromatography (HPLC) using a reverse‐phase C18 column (Supersil ODS2, 5 µm) with a UV–Vis detector, with a mobile phase of methanol and 0.1% trifluoroacetic acid (TFA) in water (9:1, v/v) at a flow rate of 1.0 mL min^−1^ and detection at 210 nm, while oxaliplatin (OXA) release was determined by measuring elemental platinum (Pt) using inductively coupled plasma (ICP) spectrometry, with Pt signals calibrated against external standards to calculate the cumulative OXA release.

### Cell Lines and Culture Conditions

Murine hepatoma cell line H22 (RRID: CVCL_H613; Cat. No. BNCC350760, BeNa Culture Collection, China), mouse embryonic fibroblasts NIH/3T3 (RRID: CVCL_ZD67; Cat. No. STCC20005P, Servicebio, China), and human coronary artery endothelial cells HCAEC (RRID: CVCL_C0EQ; Shanghai Guandao Biotechnology Co., Ltd., China) were used in this study. H22 cells were cultured in RPMI‐1640 medium (Gibco, USA), while NIH/3T3 and HCAECs were cultured in Dulbecco's Modified Eagle Medium (DMEM; Gibco, USA). All culture media were supplemented with 10% fetal bovine serum (FBS; Gibco, USA), 1% penicillin, and 1% streptomycin. Cells were maintained at 37 °C in a humidified incubator with 5% CO_2_. Mycoplasma contamination was routinely tested using a commercial Mycoplasma Stain Kit (Beyotime, China), and all cell lines were confirmed to be mycoplasma‐free.

### MTT Assay

Hydrogel cytocompatibility was assessed by monitoring cell metabolic activity. H22, NIH/3T3, and HCAEC cells were exposed to varying hydrogel concentrations for 48 h. Viability was determined using the MTT method, and absorbance values were used to compare treated versus untreated groups Equation (1).

(1) $$CellViability\left(\%\right)=\frac{A_{sample}}{A_{control}}\times 100$$

where A_sample_ and A_control_ represent the absorbance values of treated and control wells, respectively.

In addition, the cytotoxicity of oxaliplatin (OXA) and R848 toward H22 cells was assessed using the same MTT assay. Cells were incubated with serially diluted concentrations of each drug for 24 h, and the dose‐dependent inhibitory effects were analyzed by comparing cell viability across the concentration gradients.

### Animals

Female BALB/c mice (6–8 weeks old, 18–20 g) were purchased from Vital River (Beijing, China) and maintained in a specific pathogen‐free (SPF) environment. H22 tumor cells were collected from the ascitic fluid of Kunming mice and washed with PBS prior to use. For tumor implantation, 1 × 10^6^ cells suspended in 100 µL PBS were injected either subcutaneously into the right flank or orthotopically into the left hepatic lobe. In the subcutaneous model, tumor growth was monitored every other day. All procedures were approved by the Laboratory Animal Management Committee of Jilin University, China (Approval No. SYXK‐2023‐0010).

### Radiofrequency Ablation Procedure

Mice were anesthetized via intraperitoneal injection of 1% pentobarbital sodium (50 mg kg^−1^, 100 µL). After shaving and sterilizing the tumor site with 75% ethanol, radiofrequency ablation was performed using the MEDSPHERE S‐500 RF system coupled with a FATO radiofrequency generator and SVC‐500VA high‐precision voltage stabilizer. The RFA needle (ASA‐6C1T probe) was inserted along the tumor's long axis. Ablation was conducted at 70 °C for 2 min with real‐time impedance monitoring. Mice recovered on a warm pad under continuous observation.

### Anti‐Tumor Efficacy Study

BALB/c mice bearing subcutaneous H22 tumors (≈150–250 mm^3^) were randomly assigned to seven treatment groups: G1, untreated control (PBS); G2, RFA alone; G3, RFA combined with implantation of blank BI hydrogel (RFA + BI); G4, RFA followed by injection of soluble R848 and OXA (RFA + Soluble (R848 + OXA)); G5, RFA combined with BI hydrogel loaded with OXA (RFA + BI(OXA)); G6, RFA combined with BI hydrogel loaded with R848 (RFA + BI(R848)); and G7, RFA combined with BI hydrogel co‐loaded with R848 and OXA (RFA + BI(R848 + OXA)). Following treatment, hydrogel implants were placed into the ablation site in the relevant groups. To ensure fair comparison, the total drug amounts administered in the hydrogel group (G5) and the free drug control group (G4) were kept equivalent. Tumor growth was monitored regularly to evaluate the residual tumor response. Tumor volumes (V_t_) and tumor suppression rates (TSR%) were calculated using the following formulas Equations (2) and (3):

(2) Tumorvolume(Vt,mm3)=a×b2/2

where a was the major axis and b was the minor axis of the tumor.

(3) Tumorsuppressionrate(TSR%)=(Vc−Vt)/Vc×100

Here, V_c_ was the mean tumor volume (or weight) of the control group, and V_t_ was that of the treatment group at the study endpoint.

### Tumor Rechallenge Experiment

To assess treatment‐induced immune memory, a tumor rechallenge was conducted 30 days after complete tumor clearance in the RFA + BI (OXA + R848) group. Previously cured mice were re‐injected subcutaneously with 1 × 10^6^ H22 cells into the left abdominal region. Tumor growth was tracked at defined intervals. Resistance to tumor regrowth served as an indicator of long‐term immune protection. Age‐matched naive mice receiving the same tumor cell inoculation were used as controls. Tumor sizes were monitored periodically, and animals were sacrificed once tumors exceeded 2000 mm^3^.

### Bilateral Tumor Model and Treatment Protocol

To assess systemic antitumor immunity, a bilateral H22 tumor model was generated using BALB/c mice. Briefly, 1 × 10^6^ H22 cells in 100 µL PBS were inoculated subcutaneously into the left flank. When tumors reached ≈150 mm^3^ on average, animals were randomly assigned to different treatment groups.

1. PBS (control)
2. RFA (radiofrequency ablation only)
3. RFA + BI (blank biopolymer implant)
4. RFA + Soluble (OXA + R848)
5. RFA + BI (OXA)
6. RFA + BI (R848)
7. RFA + BI (R848 + OXA)

Treatments were administered immediately following RFA, as per group assignments.

After the primary tumor treatment, a secondary incubation was performed by injecting 1 × 10^6^ H22 cells subcutaneously into the right flank of the same mice.

Mice were euthanized when the tumor volume on either flank exceeded 2000 mm^3^, or if they met humane endpoints as defined by institutional animal care guidelines.

### Surgical Protocol for Hepatic Tumor Induction and RFA‐BI Treatment in BALB/c Mice

To establish an orthotopic hepatocellular carcinoma model, female BALB/c mice (6‐8 weeks old) were anesthetized with 2% isoflurane and maintained on a heated surgical pad throughout the procedure. Under aseptic conditions, abdominal hair was removed, and the skin was disinfected sequentially with povidone‐iodine and 70% ethanol, repeated three times. A horizontal incision ≈1 cm in length was made below the left costal margin to expose the left liver lobe without entering the thoracic cavity. The peritoneum was carefully lifted and incised to minimize visceral injury. Using a fine 27–29G needle, 5 × 10^5^ H22 hepatoma cells in 20 µL PBS were slowly injected tangentially a few millimeters beneath the liver capsule at a shallow angle to deliver the cells into the subcapsular region while avoiding capsule penetration. A pale bleb visible on the liver surface indicated successful intraparenchymal delivery. After injection, the needle was left in place for several seconds before slow withdrawal to minimize backflow. A sterile cotton swab was then applied to the injection site for ≈15 s to achieve hemostasis and prevent leakage. The incision was temporarily closed, and the mice were returned to their cages for tumor establishment.

Seven days post‐injection, when the tumors were established, mice were anesthetized again, and the prior surgical site was reopened to expose the tumor‐bearing liver lobe. Radiofrequency ablation (RFA) was performed using a standard protocol to ablate the tumor tissue. Immediately after RFA, mice received either an injection of PBS or biopolymer implants loaded with immunomodulators directly into the ablation site. The hydrogel was placed carefully onto the treated liver surface to ensure local retention. Finally, the abdominal wall and skin were sutured in layers, and mice were monitored postoperatively until full recovery.

### Flow Cytometry

After treatment, tumors and spleens were collected for cellular analysis. Tumor samples were enzymatically digested at 37 °C for 1 h using a mixture of collagenase, hyaluronidase, and DNase I, followed by mechanical dissociation and filtration through a 70 µm strainer. The resulting single‐cell suspension was processed and resuspended in FACS buffer. Spleen tissues were similarly dissociated, treated with red blood cell lysis buffer, and washed. Both tumor‐ and spleen‐derived cells were labeled with fluorescent antibodies targeting CD8⁺ T cells, NK cells, and dendritic cells (Table S1, Supporting Information). Flow cytometric analysis was performed on a BD FACS Canto II system, and results were processed using FlowJo.

### Histological and Immunofluorescence Staining

Tumor tissues were preserved in 4% paraformaldehyde, paraffin‐embedded, and sectioned at 5 µm thickness. H&E staining was utilized to evaluate histological features and detect inflammation or tissue damage. For immunofluorescence, sections were probed with antibodies against Ki67, TUNEL, CD4, and CD8, followed by labeling with fluorophore‐linked secondary antibodies. Imaging was conducted using a fluorescence microscope.

### RNA Sequencing and Analysis

At the study endpoint, tumor tissues were collected, frozen in liquid nitrogen, and stored at −80 °C. RNA was extracted using Trizol, and quality was assessed by Bioanalyzer. Poly(A)‐selected RNA libraries were prepared and sequenced on the Illumina HiSeq 4000 platform. Differential expression analysis was performed using DESeq2, with thresholds set at fold change > 2 and *P* < 0.05. Pathway enrichment was conducted based on KEGG annotations.

### RNA Extraction, Reverse Transcription, and Quantitative RT‐qPCR

Total RNA was isolated from tumor tissues using TRIzol reagent according to standard protocols. Reverse transcription and quantitative real‐time PCR (RT‐qPCR) were performed with a one‐step RT‐qPCR kit. Gene‐specific primers were designed for the following targets: H2BI (forward: 5′‐AGATCCCCCAAAGGCACATG‐3′, reverse: 5′‐ATTCAACTGCCAGGTCAGGG‐3′), Map2k4 (forward: 5′‐AGCATGCAGGGCTTTCAGAT‐3′, reverse: 5′‐GTGTGGGTTCTGGACTCCTG‐3′), Map2k3 (forward: 5′‐CGGCATCACCATGATCGAGA‐3′, reverse: 5′‐GCTCTGCAGGGTTCTTCCTT‐3′), Akt1 (forward: 5′‐CGCTTCTATGGTGCGGAGAT‐3′, reverse: 5′‐GTTCTCCAGCTTCAGGTCCC‐3′), and Pik3cb (forward: 5′‐CTGATTTTACGGCGGCATGG‐3′, reverse: 5′‐TGAGGGCCTCGTCAAACTTC‐3′). RT‐qPCR was performed under standard thermal cycling conditions, and relative gene expression levels were calculated using the comparative Ct (ΔΔCt) method.

### Statistical Analysis

Quantitative results were expressed as mean ± standard deviation (SD). Two‐tailed unpaired Student's t‐tests were used for comparisons between two groups, and one‐way ANOVA was applied for comparisons among multiple groups. Survival data were analyzed by the Kaplan‐Meier method and compared using the log‐rank test. Statistical analysis was performed using GraphPad Prism 10 and Microsoft Excel 365. A *P*‐value < 0.05 was considered statistically significant.

## Conflict of Interest

The authors declare no conflict of interest.

## Supporting information



Supporting Information

## Data Availability

The data that support the findings of this study are available from the corresponding author upon reasonable request.
