# Supplementary material for: Hyaluronic Acid Hydrogel Implants for Sustained Release of Oxaliplatin and Resiquimod to Prevent Hepatocellular Carcinoma Recurrence Post‐Radiofrequency Ablation
Source: Adv Sci (Weinh). 2025 Sep 30;12(47):e09309. doi: 10.1002/advs.202509309 (PMC12713106; doi:10.1002/advs.202509309)
Supplement: Supplementary file 1 — Supporting Information [file ADVS-12-e09309-s001.docx]

Supporting information

Hyaluronic acid hydrogel implants for sustained release of oxaliplatin and resiquimod to prevent hepatocellular carcinoma recurrence post-radiofrequency ablation

Yuezhan Shan^1^, Hongyu Chu^1^, Sheyu Ye^5^, Guofeng Ji^3^, Jiayi Zhao^1^, Xinghui Si^4^, Yumin Zhong^1^, Youmao Tao^1,*^, Jingwei Shi^2,*^, Xuedong Fang^1,*^

*^1^ Y.Z. Shan, H.Y. Chu, J.Y. Zhao, Y.M. Zhong, Y.M. Tao, X.D. Fang*

*Department of Gastrointestinal and Colorectal Surgery, China-Japan Union Hospital of Jilin University, Changchun, 130033, China*

*^2^ J.W. Shi*

*Department of Laboratory Medicine Center, China-Japan Union Hospital, Jilin University, Changchun, 130033, China*

*^3^ G.F. Ji*

*Department of General Surgery, Xuanwu Hospital, Capital Medical University, Beijing 100053, China*

*^4^ X.H. Si*

*Key Laboratory of Polymer Ecomaterials, Changchun Institute of Applied Chemistry, Chinese Academy of Sciences, Changchun 130022, China*

*^5^ S.Y. Ye*

*North Sichuan Medical College, Sichuan 637199, China*

E-mail: taoym@jlu.edu.cn (Y. Tao); [shijingwei@jlu.edu.cn (J, Shi);](mailto:shijingwei@jlu.edu.cn;) fangxd@jlu.edu.cn (X. Fang)

#
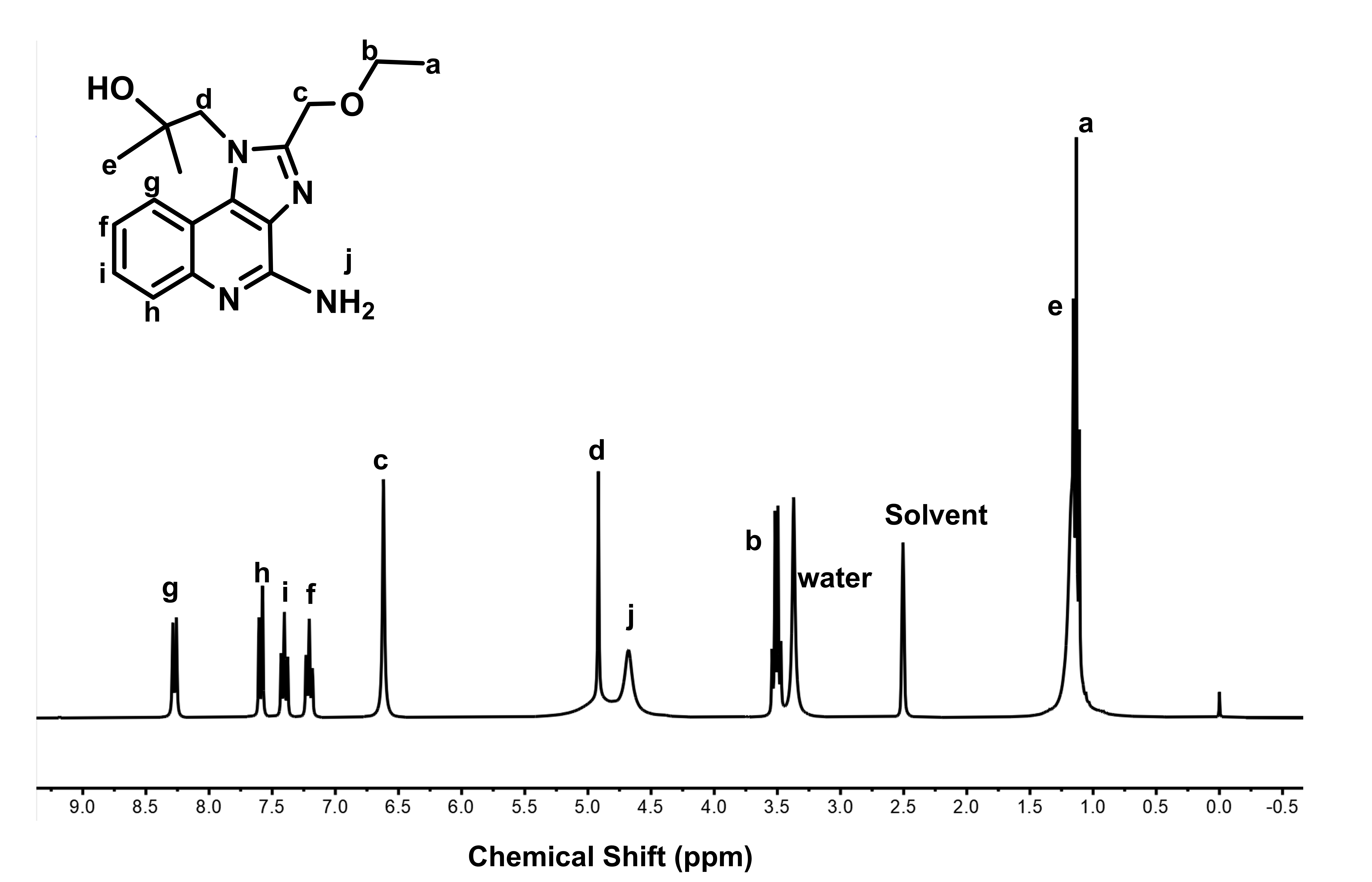


# Figure S1 ^1^H NMR spectrum of R848 in DMSO-*d_6._*


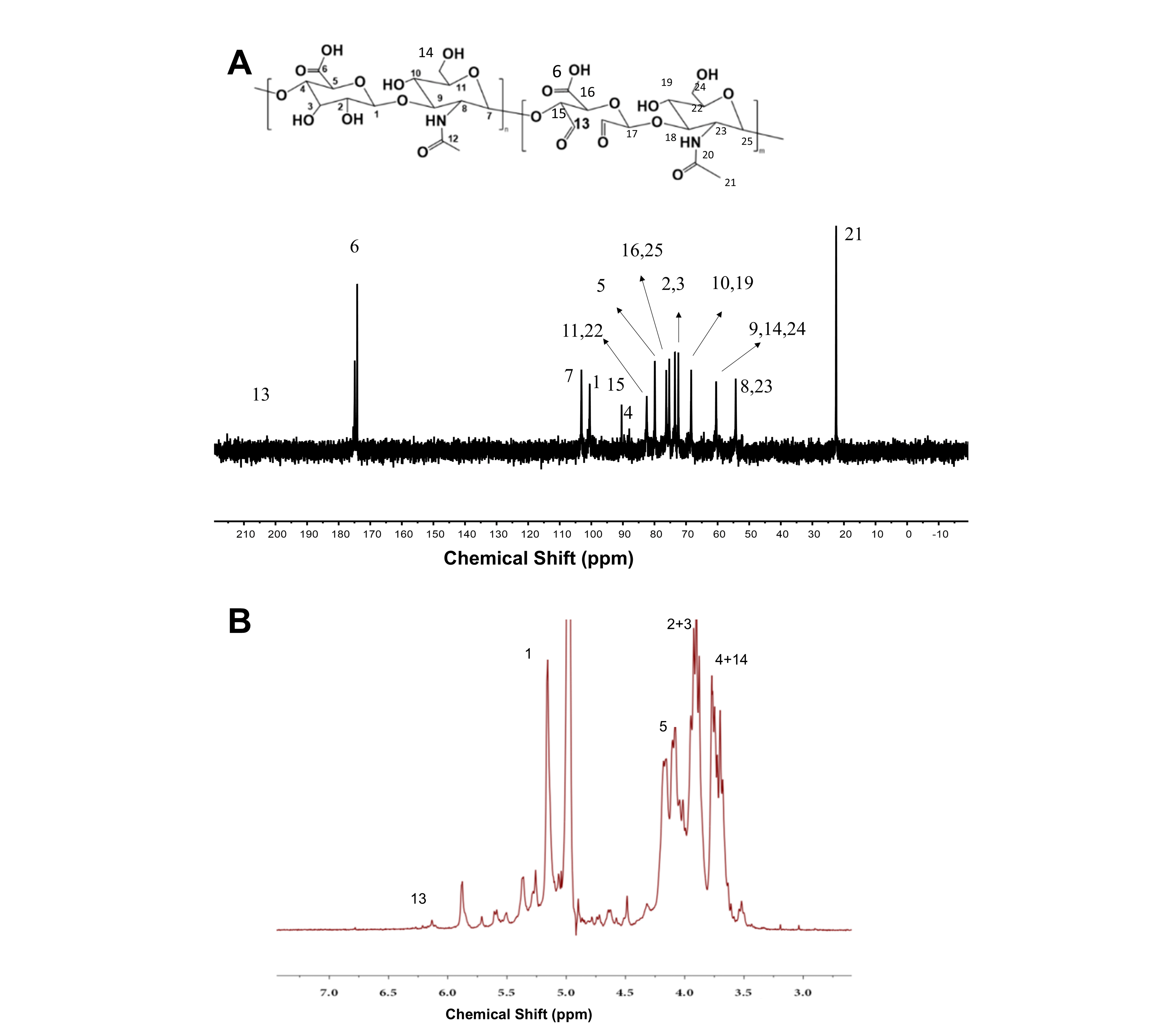


# Figure S2 ^13^C NMR (A) and ^1^H NMR (B) spectrum of oxidized hyaluronic acid in D_2_O.

**
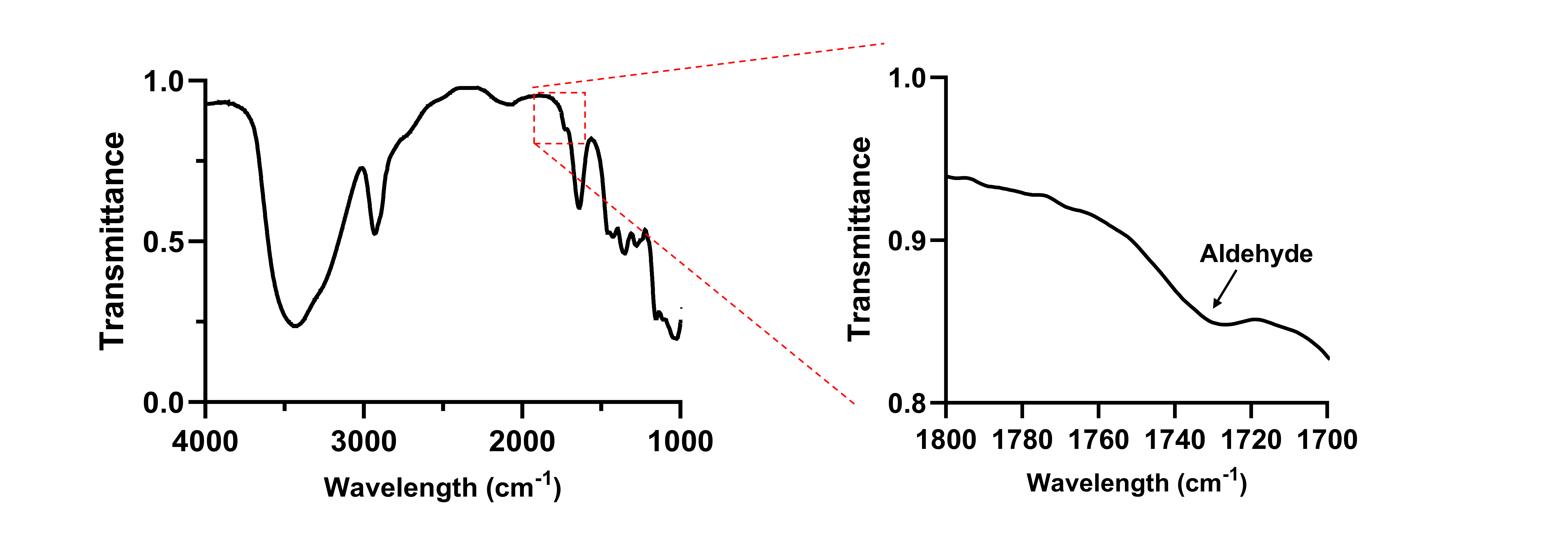
**

# Figure S3 FTIR spectrum of oxidized hyaluronic acid.

**
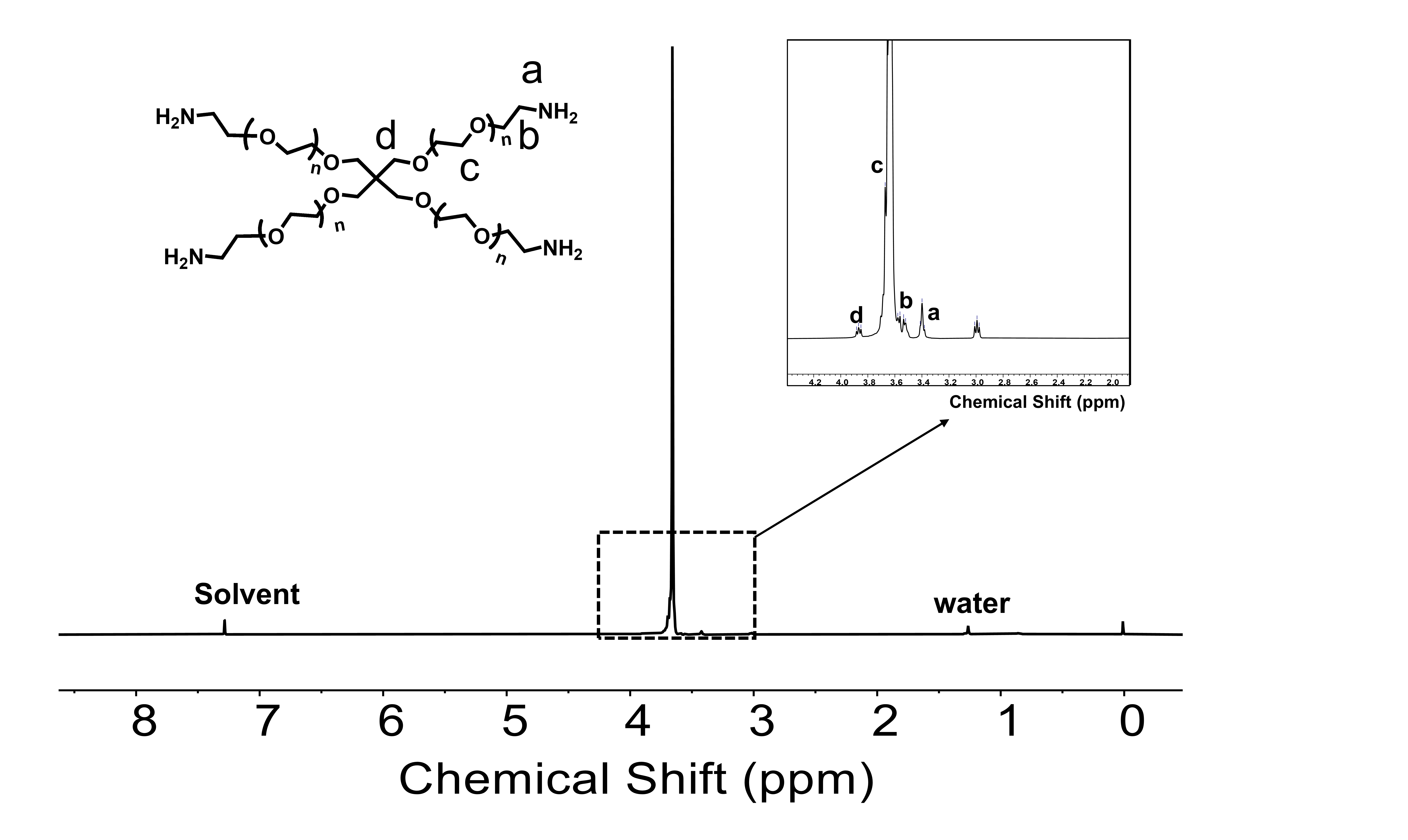
**

# Figure S4 ^1^H NMR spectrum of 4-arm-PEG-NH_2_ (10 kDa) in CDCl_3_.


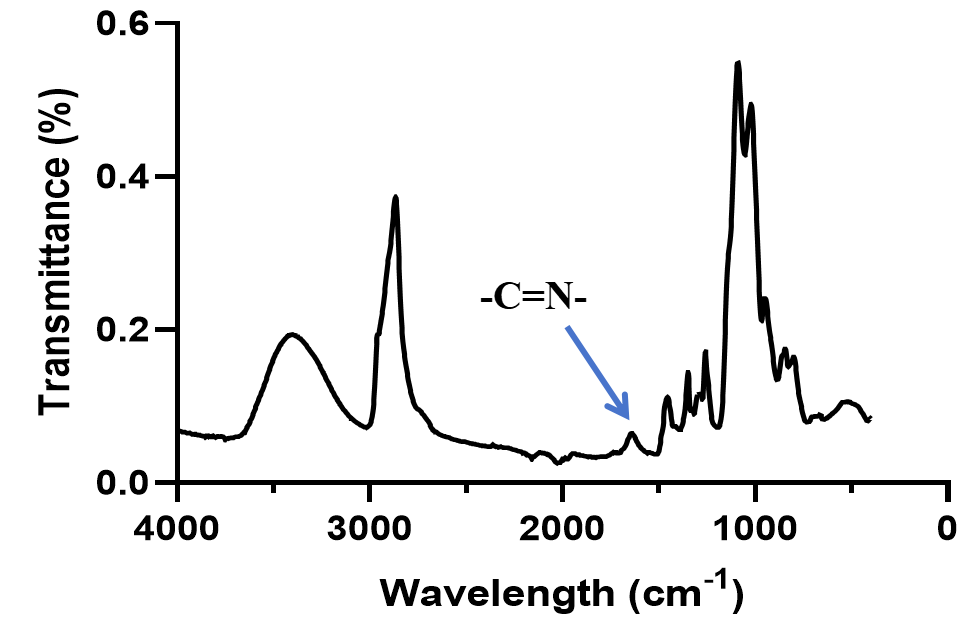


**Figure S5**. FTIR spectrum of the freeze-dried hydrogel BI.

**Figure S6.** Tensile strength of BI between mouse skins and BI.


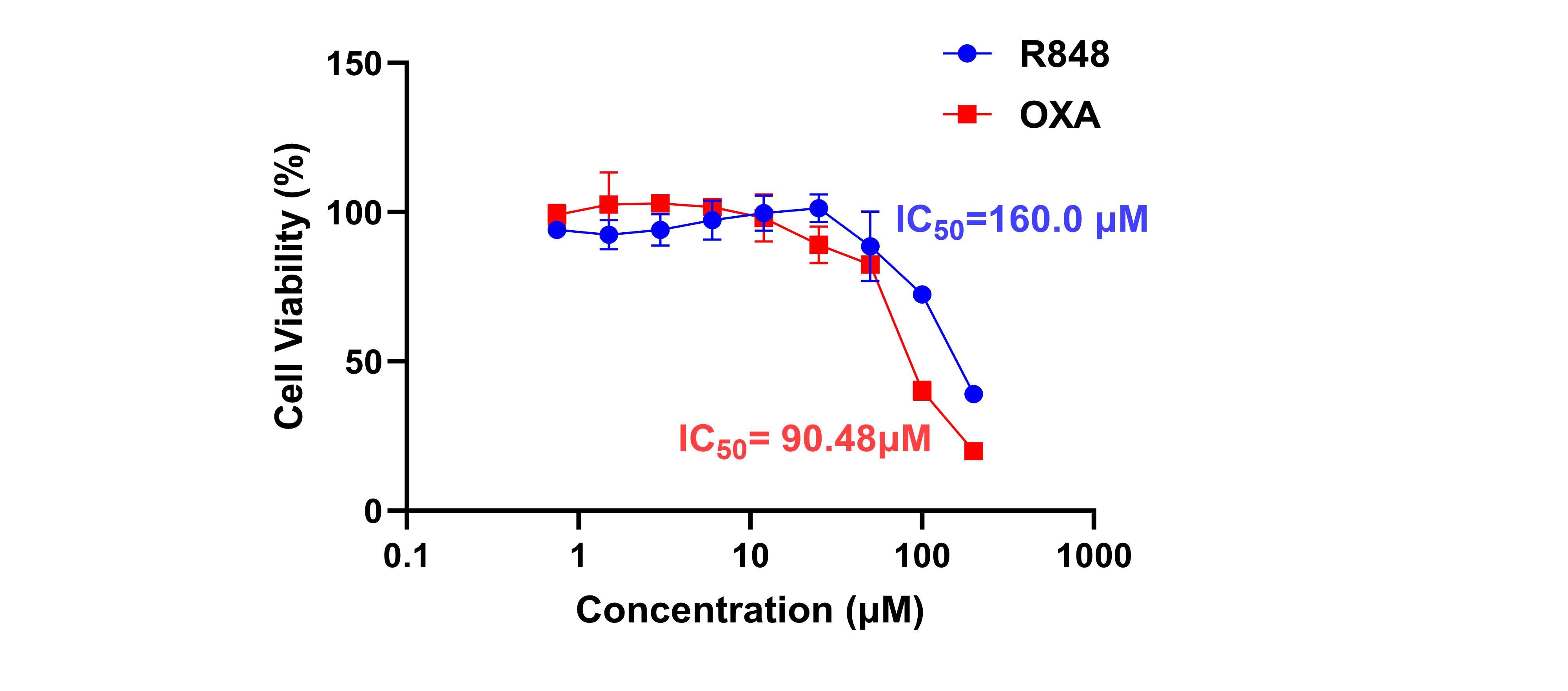


**Figure S7.** Cell viability of H22 cells treated with gradient concentrations of R848 and OXA for 24 h, as determined by the MTT assay. Data are presented as mean ± SD (n = 3).


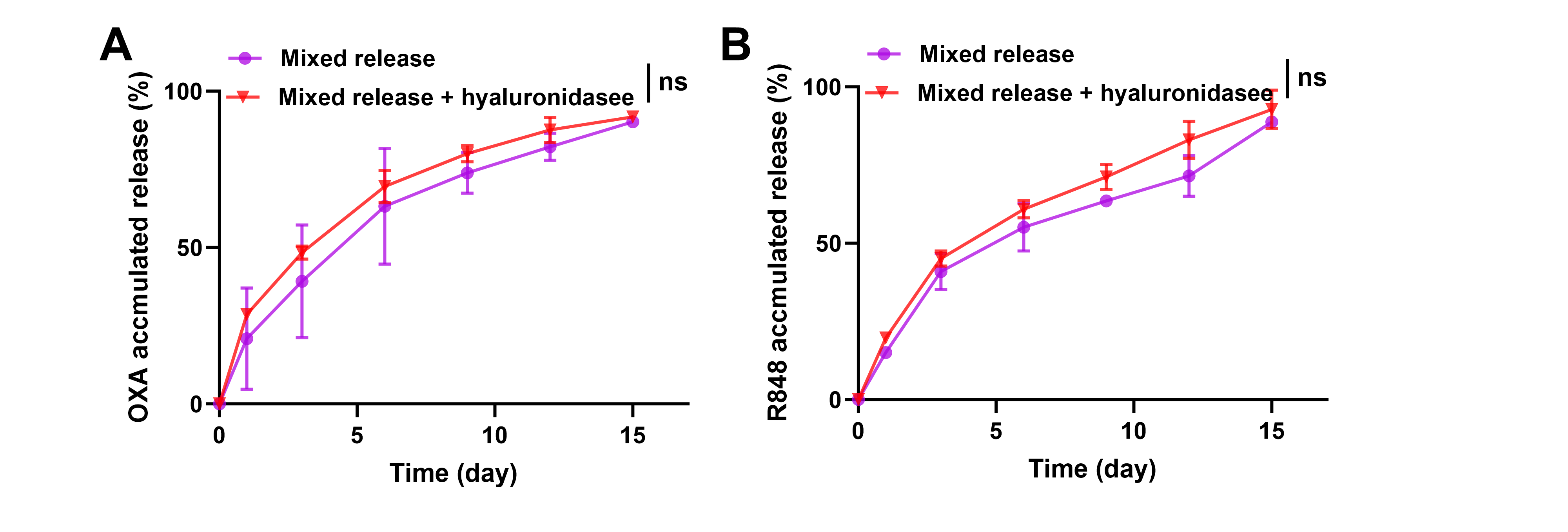


**Figure S8** Cumulative release profiles of OXA (A) and R848 (B) from BI hydrogel in PBS with and without 5 mg/mL HAase (n = 3).

**
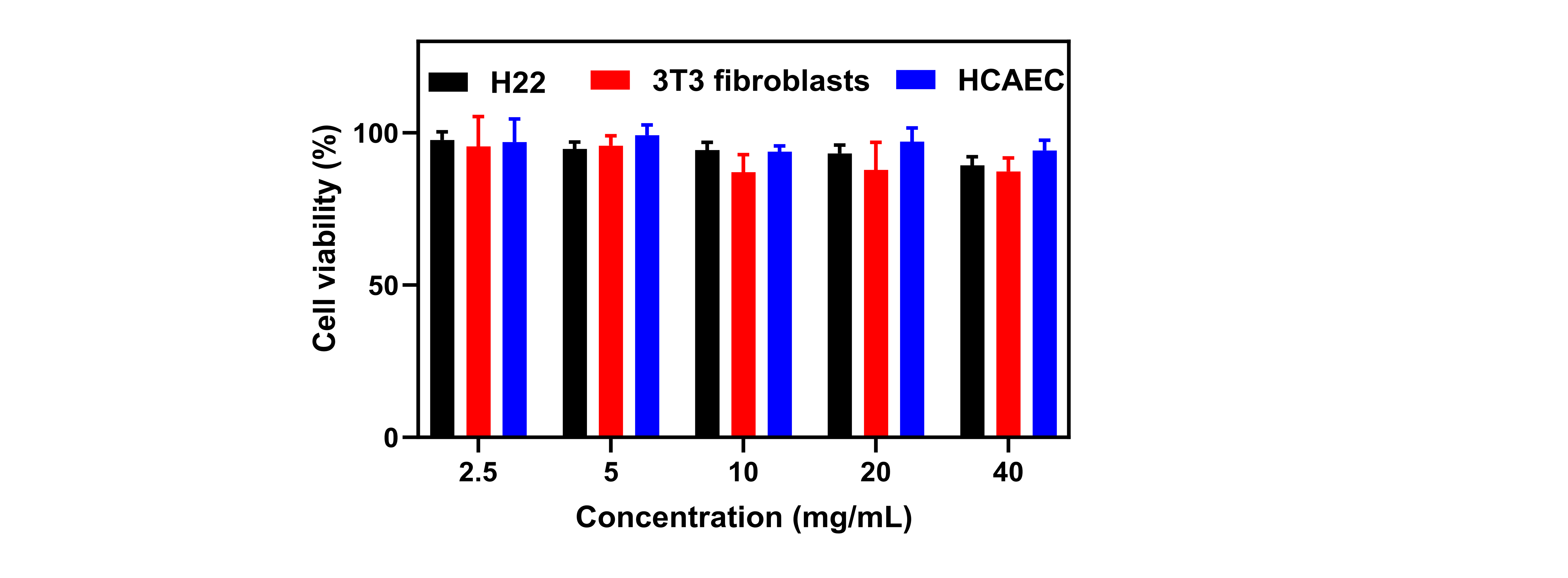
**

# Figure S9. Cell viability of different cell lines after incubation with the implant for 48 h (n = 5).

#
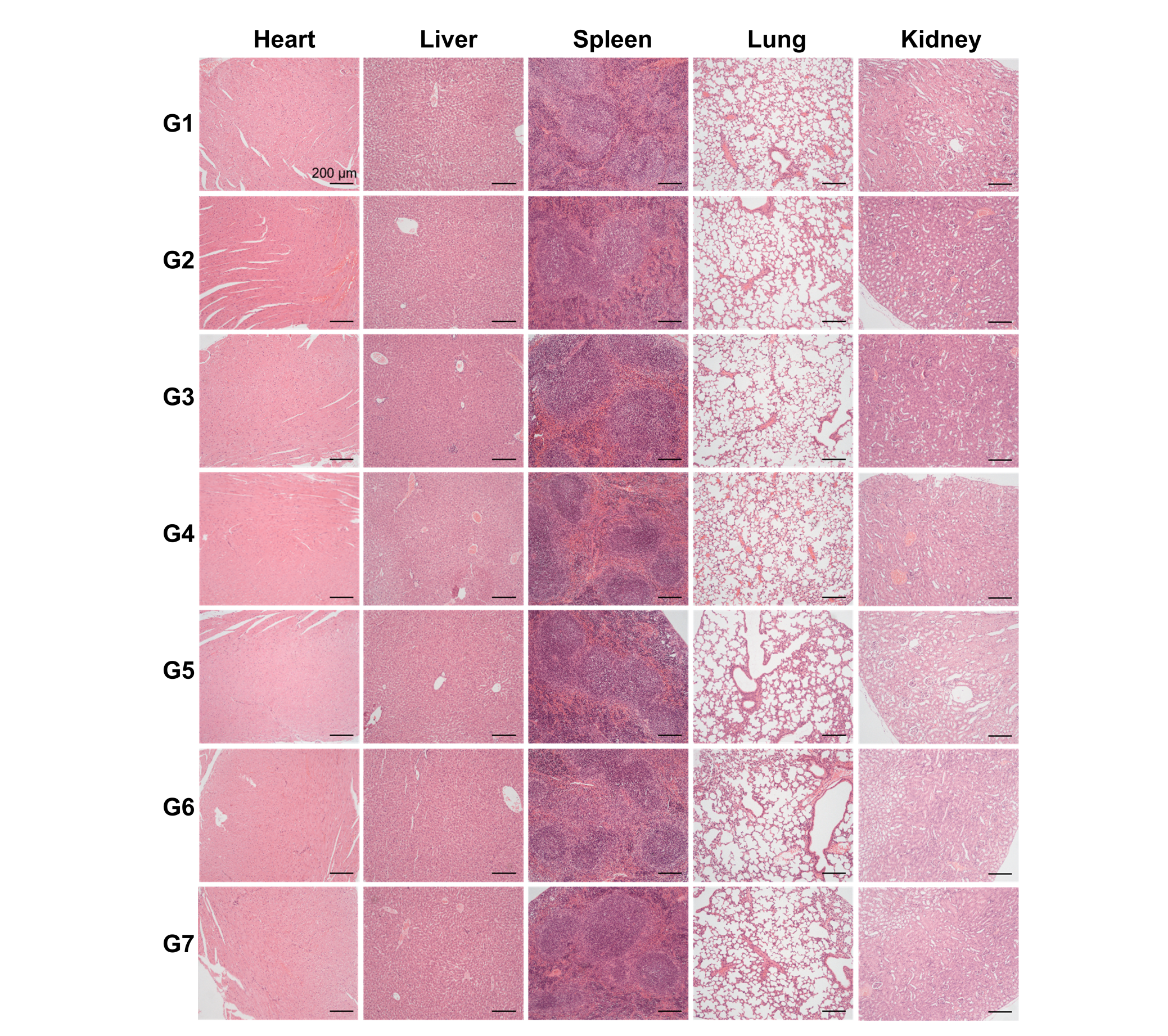
Figure S10 H&E staining images of normal tissues after various treatments. Scale bar: 200 μm. G1: PBS; G2: RFA; G3: RFA + BI; G4: RFA + Soluble(R848 + OXA); G5: RFA + BI(OXA); G6: RFA + BI(R848); G7: RFA + BI(R848 + OXA).

**
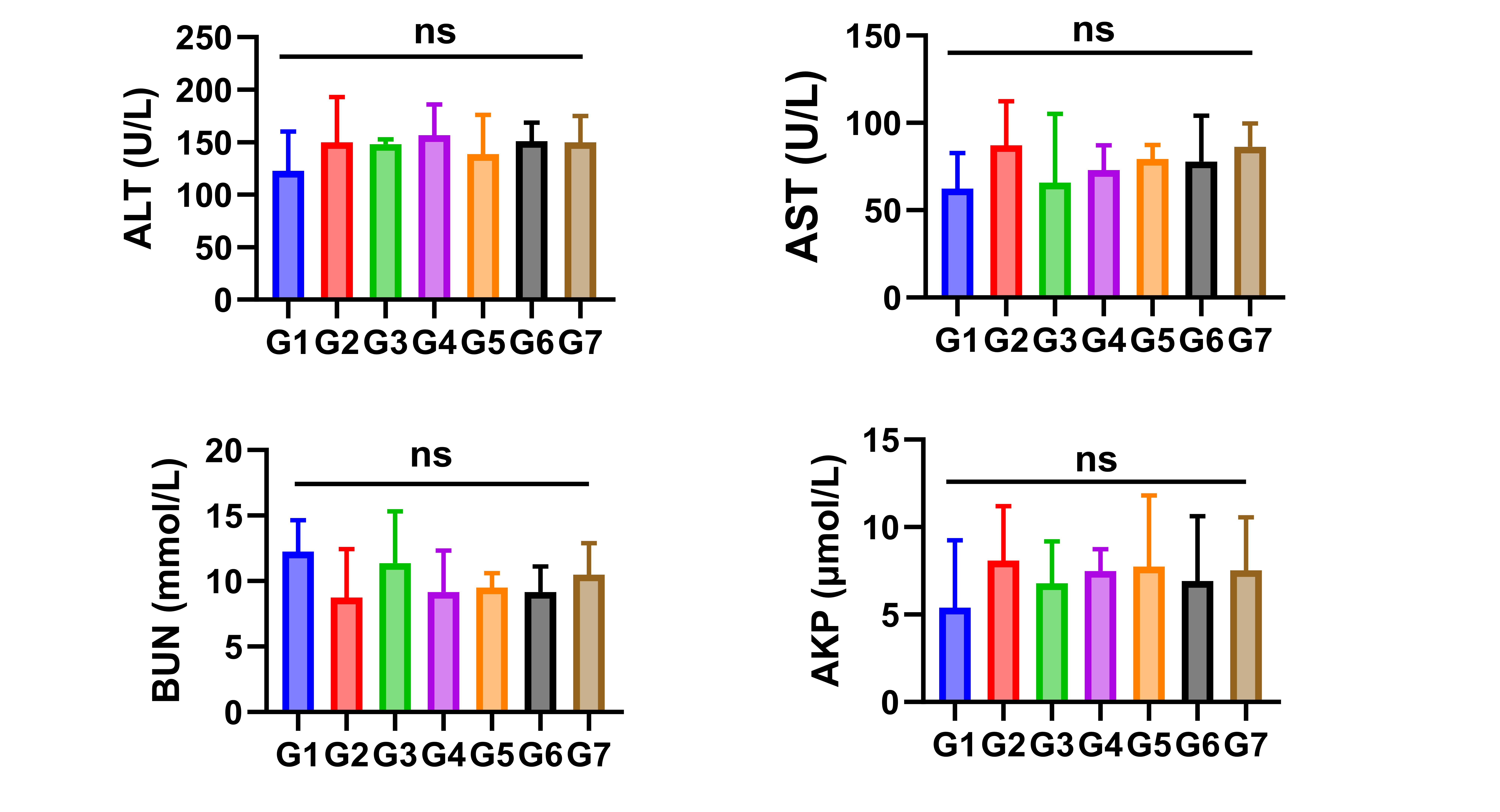
**

# Figure S**11** Hematological analysis of the H22 tumor model following treatment with PBS G1: PBS;G2: RFA; G3: RFA + BI; G4: RFA + Soluble(R848 + OXA); G5: RFA + BI(OXA);G6: RFA + BI(R848);G7: RFA + BI(R848 + OXA).

**
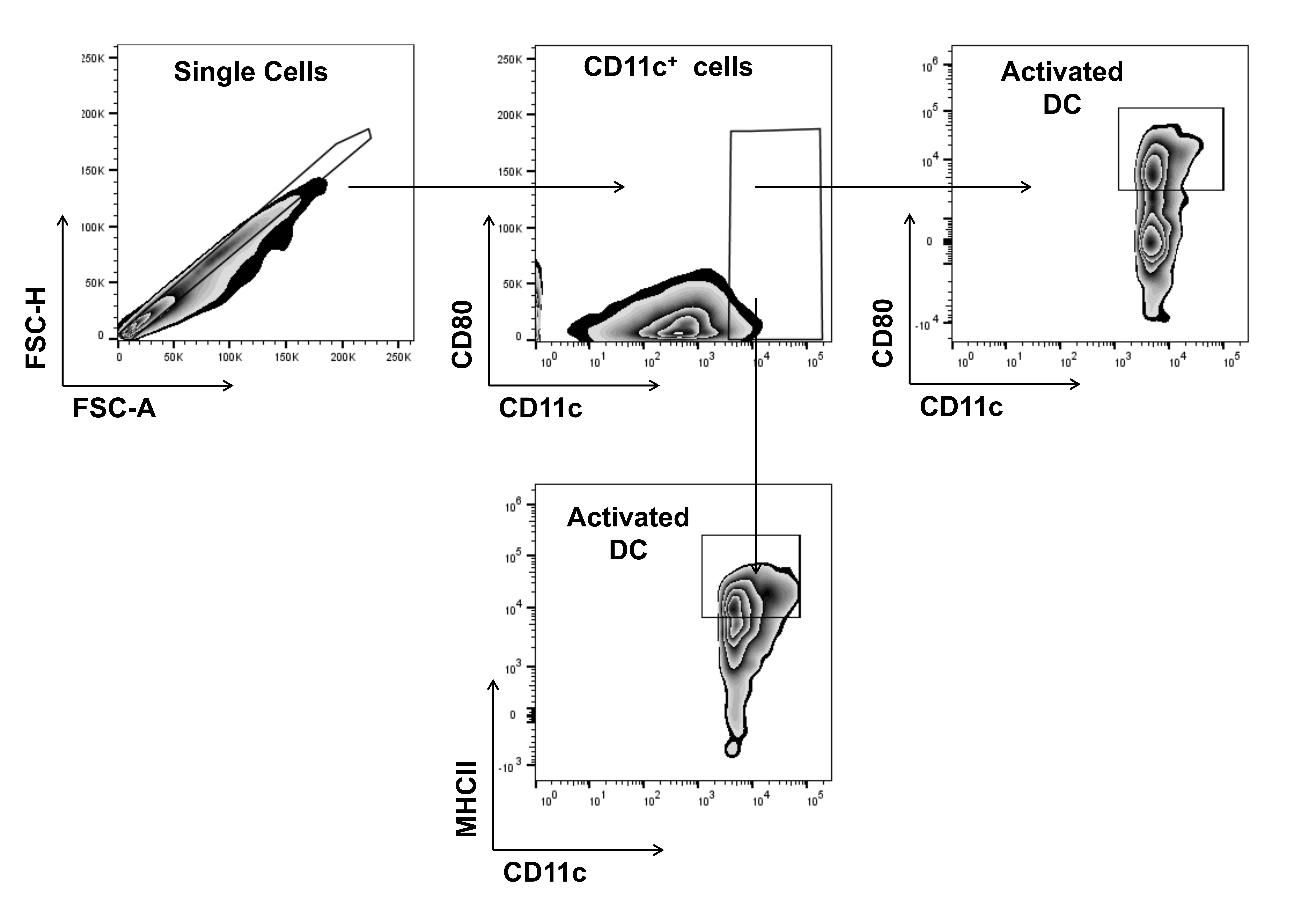
**

# Figure S12 Gating strategy for activated dendritic cells in tumor tissues. Activated DCs were identified as CD11c^+^CD80^+^ and CD11c^+^MHCII^+^ populations.

**
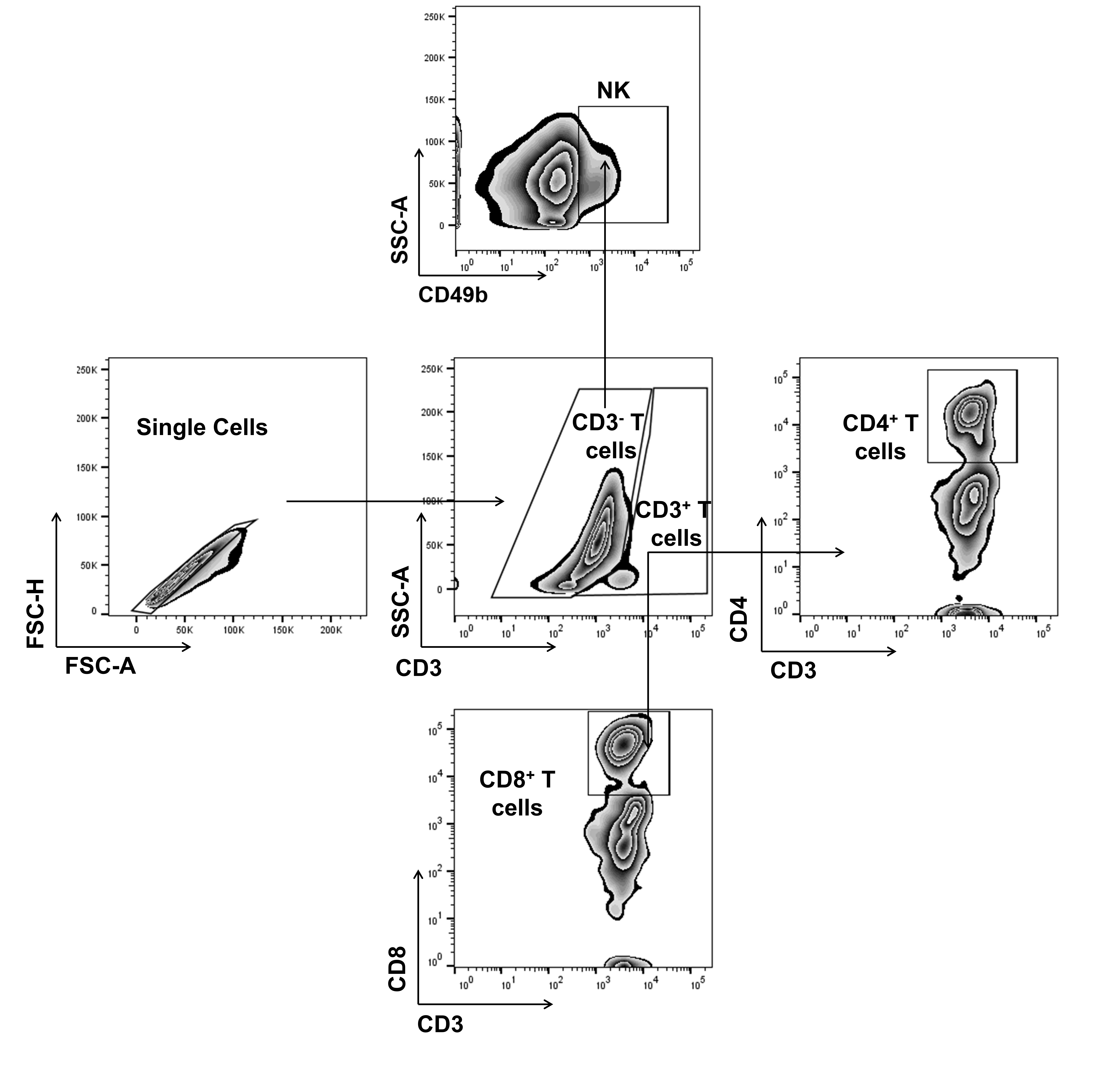
**

# Figure S13 Gating strategy for tumor-infiltrating T cells and NK cells. CD4^+^ and CD8^+^ T cells were identified as CD3^+^CD4^+^ and CD3^+^CD8^+^ populations, respectively; NK cells were gated as CD3^-^NK1.1^+^.

*
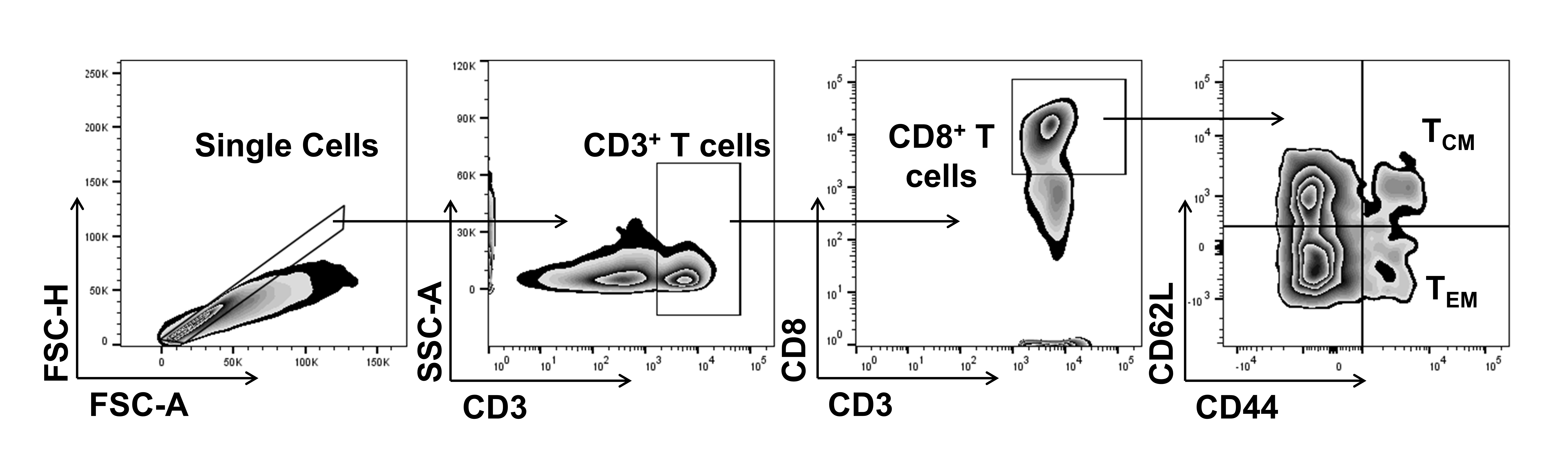
*

# Figure S14 Gating strategy for central memory (CD44^+^CD62L^+^) and effector memory (CD44^+^CD62L^-^) CD8^+^ T cells in spleen.

# Table S1 Antibodies used in the experiment

| Antibodies | Company | Catalog | Application |
| --- | --- | --- | --- |
| FITC anti-mouse CD3 Antibody | BioLegend | 100203 | flow |
| PE/Cy7 anti-mouse CD4 Antibody | BioLegend | 100422 | flow |
| APC anti-mouse CD8a Antibody | BioLegend | 100712 | flow |
| APC/Cy7 anti-mouse CD49b Antibody | BioLegend | 108724 | flow |
| PE anti-mouse CD44 Antibody | BioLegend | 103007 | flow |
| APC/Cy7 anti-mouse CD62L Antibody | BioLegend | 104418 | flow |
| APC/Cy7 anti-mouse CD11b Antibody | BioLegend | 101226 | flow |
| APC anti-mouse CD80 Antibody | BioLegend | 104714 | flow |
| PE anti-mouse CD11c Antibody | BioLegend | 117308 | flow |
| APC/Cy7 anti-mouse I-A/I-E (MHCII) Antibody | BioLegend | 107628 | flow |
